# Biomaterial‐based gene therapy

**DOI:** 10.1002/mco2.259

**Published:** 2023-06-03

**Authors:** Yi Yu, Yijun Gao, Liming He, Bairong Fang, Wenhui Ge, Pu Yang, Yikun Ju, Xiaoyan Xie, Lanjie Lei

**Affiliations:** ^1^ Department of Stomatology The Second Xiangya Hospital Central South University Changsha China; ^2^ Department of Stomatology Changsha Stomatological Hospital Changsha China; ^3^ Department of Plastic and Aesthetic (Burn) Surgery The Second Xiangya Hospital Central South University Changsha China; ^4^ State Key Laboratory of Bioelectronics, School of Biological Science and Medical Engineering Southeast University Nanjing China

**Keywords:** biomaterial, gene therapy, nonviral vector, viral vector

## Abstract

Gene therapy, a medical approach that involves the correction or replacement of defective and abnormal genes, plays an essential role in the treatment of complex and refractory diseases, such as hereditary diseases, cancer, and rheumatic immune diseases. Nucleic acids alone do not easily enter the target cells due to their easy degradation in vivo and the structure of the target cell membranes. The introduction of genes into biological cells is often dependent on gene delivery vectors, such as adenoviral vectors, which are commonly used in gene therapy. However, traditional viral vectors have strong immunogenicity while also presenting a potential infection risk. Recently, biomaterials have attracted attention for use as efficient gene delivery vehicles, because they can avoid the drawbacks associated with viral vectors. Biomaterials can improve the biological stability of nucleic acids and the efficiency of intracellular gene delivery. This review is focused on biomaterial‐based delivery systems in gene therapy and disease treatment. Herein, we review the recent developments and modalities of gene therapy. Additionally, we discuss nucleic acid delivery strategies, with a focus on biomaterial‐based gene delivery systems. Furthermore, the current applications of biomaterial‐based gene therapy are summarized.

## Graphical abstract

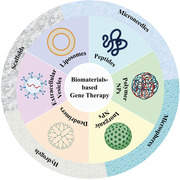

## 1 INTRODUCTION

Gene therapy involves the introduction of a target gene into a target cell. Subsequently, the target gene becomes part of the hereditary material of the host cell, while the expression of the target gene eventually contributes to host disease of the host.^1^ The origin of gene therapy can be traced back to the idea of gene exchange and optimization, which was introduced by Joshua Lederberg in 1963. In 1972, Friedmann et al.^2^ published a prospective review in *Science*, which predicted the potential of gene therapy for alleviating hereditary diseases caused by a single gene. Gene therapy was converted from concept to clinical trials in the early 1990s, with the first gene therapy clinical trial being led by William French Anderson, MD, in 1990 for severe combined immunodeficiency.^3^ The success of this clinical trial then set off a subsequent wave of further gene therapy clinical trials. However, early clinical studies encountered inefficacy and serious adverse effects, some of which even led to death. For example, an 18‐year‐old trial participant died in 1999 after receiving experimental gene therapy for ornithine transcarbamylase deficiency. This tragedy marked a low point for gene therapy clinical trials and prompted scientists to conduct deeper basic research into gene transfer vectors, target cells and tissues, and knowledge of disease biology.^4^ Viral vector technology can only be used to add genes, whereas gene editing technology can add, knock down, or correct genes. In 1996, the zinc finger enzyme gene editing technology was invented.^5^ Furthermore, the advent of clustered regularly interspaced short palindromic repeats (CRISPR/Cas9) gene editing technology in 2012 revolutionized the field of gene therapy by simplifying the workload while reducing the costs of gene editing.^6^ Subsequently, scientists discovered other Cas enzymes with unique properties and have continued to develop novel gene editing methods that can edit multiple loci in the genome simultaneously, such as Cas Hybrid for Multiplexed Editing and screening Applications.^7^, ^8^ The United States Food and Drug Administration (US FDA) approved the first gene therapy products in 2017, which included chimeric antigen receptor (CAR)‐T cell therapy products (Kymriah and Yescarta) for the treatment of refractory B‐cell malignancies^9^ and an Adeno‐associated virus (AAV) vector (Luxturna) for the in vivo treatment of inherited retinal diseases (IRDs).^10^ Improvements in scientific research, safety, gene transfer efficiency, and further maturation of infusion techniques in gene therapy have ultimately led to significant clinical advances.^11^ Gene therapy can treat both hereditary diseases and complex acquired diseases such as cancer, heart disease, neurodegenerative diseases, and infectious diseases.^12^ The basic strategies of existing gene therapies comprise gene replacement, addition, intervention, suicide therapy, and immunotherapy. Nucleic acid molecules alone are not easily absorbed by cells and are prone to degradation and clearance in the in vivo environment. Therefore, they require carriers for cellular delivery.^13^ However, a major obstacle to the clinical use of gene therapy is the lack of safe and effective delivery vectors.

The successful transfer of exogenous genes is essential for gene therapy. This review discusses the traditional basic techniques of gene transfer, including physical, viral, and nonviral vector‐mediated methods. Various viral vectors have been extensively studied in clinical trials and research on therapies involving gene delivery. However, challenges in gene recombination with wild‐type viruses, the high cytotoxicity and immunogenicity of viral vectors, limited cargo loading capacities, and production difficulties have greatly limited their application. Nonviral vector systems can enhance biological stability and the efficiency of intracellular gene delivery techniques without the disadvantages of viral vectors. Nonviral vectors are therefore considered good substitutes for viral vectors.

**FIGURE 1:**
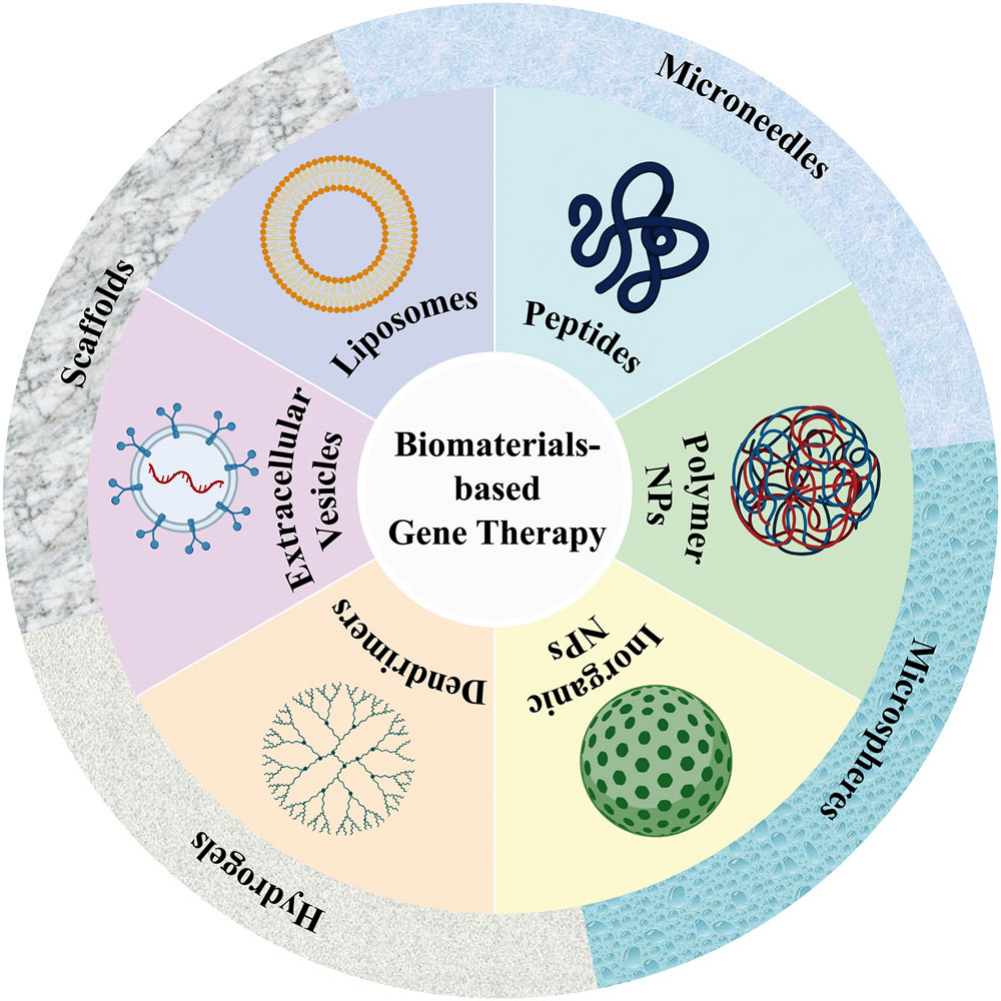
Overview of biomaterial‐based delivery systems for gene therapy.

**FIGURE 2:**
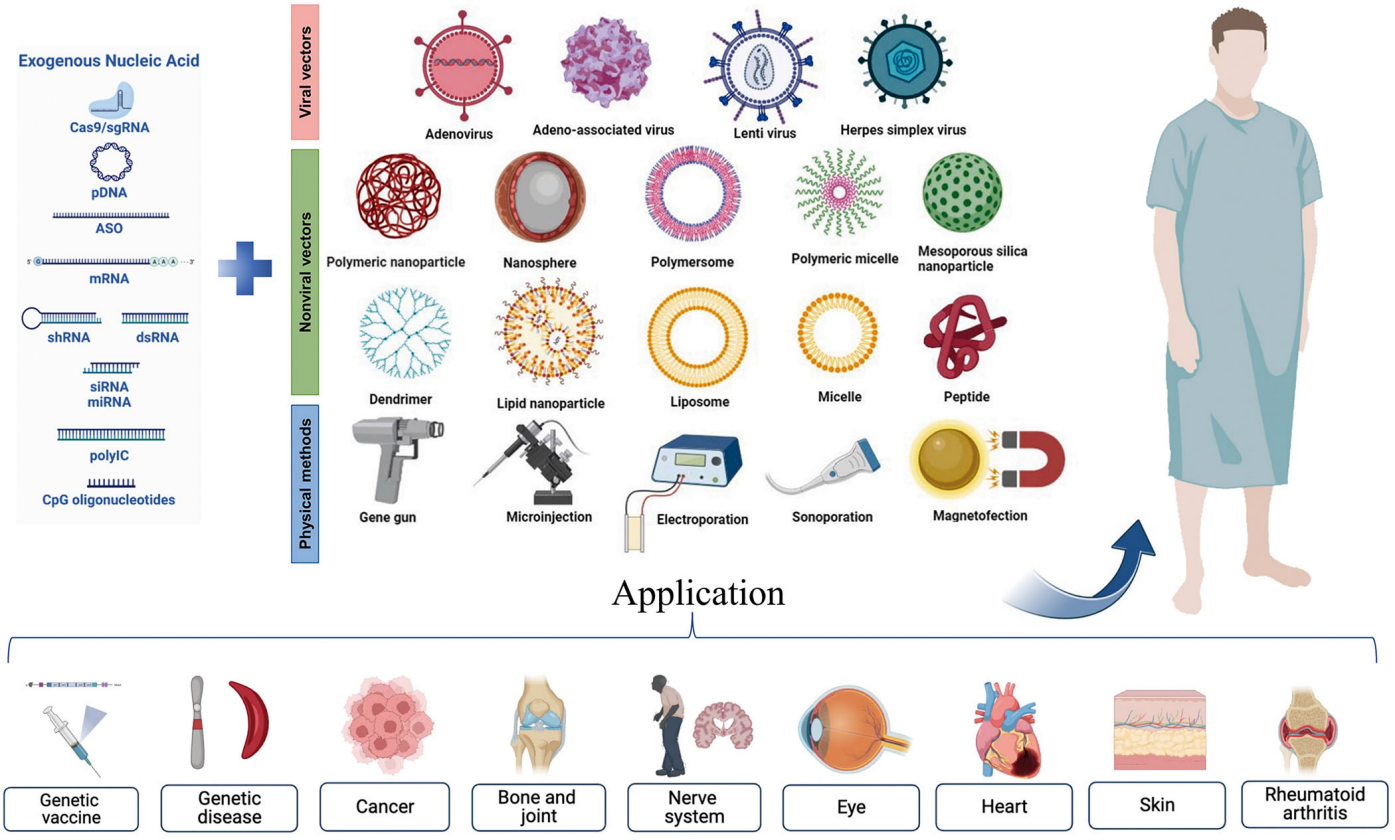
A review of biomaterial‐based gene therapy. Exogenous nucleic acid entering the human body combined with different types of viral vectors, nonviral vectors, and physical methods. The Exogenous nucleic acid illustration is part of a figure reproduced with permission from Ref. 114, Copyright 2022 © Elsevier B.V. The different types of viral vectors, nonviral vectors, and physical methods is reproduced with permission from Ref. 13, Copyright 2022 © Elsevier B.V. The application in patients with different diseases is created with BioRender.com.

With the emergence of surface‐modified or functional materials, nonviral gene delivery vectors based on biomaterials have developed rapidly. Nonviral vectors can be summarized as liposomes and their derivatives, cationic polymers, dendrimers, peptides, and cell derivatives. These vectors can be used in combination with slow‐release delivery systems, such as microneedles, microspheres, hydrogels, and scaffolds, to provide longer‐lasting effects and minimize potential toxicity when used systemically (Figure 1). Biomaterial‐based delivery systems have sufficient packaging capacities and multiple functions to continuously and locally deliver drugs to their target sites. Natural biomaterials have valuable properties, including biocompatibility, biodegradability, and low toxicity, and are easily metabolized by host tissues. Synthetic biomaterials have the advantages of adjustable mechanical properties and ease of large‐scale manufacturing. This paper focuses on biomaterial‐based delivery systems, reviews the systems used or have potential applications in gene therapy and vaccines, to provide a theoretical basis for the development of biomaterial‐based exogenous gene delivery systems based on biomaterials (Figure 2).

## 2 GENE THERAPY

### 2.1 Theoretical basis of gene therapy

Gene therapy research assesses three main approaches: (i) introduce exogenous genes into diseased cells to produce normal gene expression products to further supplement missing or loss‐of‐function proteins (i.e., by upregulating gene expression); (ii) downregulating gene expression by using small interfering RNA (siRNA), antisense oligonucleotides (ASOs), short hairpin RNA (shRNA), or microRNA (miRNA); and (iii) editing mutated genes using zinc finger nucleases, transcription activator‐like effector nucleases, or CRISPR/Cas9 technology, resulting in gain or loss‐of‐function.^14^, ^15^, ^16^, ^17^

Gene therapies can be classified as germline or somatic, depending on the cells targeted. Germline gene therapies (GGTs) target germ cells; therefore, GGTs can not only treat genetic diseases in the first generation of offspring but also pass on new genes to future generations, so genetic diseases can be mitigated. A form of GGT called “mitochondrial replacement therapy” (MRT) has been developed and acts by replacing mutated mitochondrial DNA (mtDNA) in mutant carrier oocytes with donated, mutation‐free counterparts, allowing women with mtDNA mutations to conceive healthy children. The first successful human case involving MRT was reported in Mexico.^18^ CRISPR/Cas9 technologies can be used to edit germline genes; however, there are many technical problems with CRISPR/Cas9 gene‐edited embryos, such as mosaicism, off‐target effects, and chromosomal structural abnormalities.^19^ There are also ethical issues surrounding GGTs, since they can have unpredictable consequences.

Somatic gene therapies, which comprise the focus of this study, can be divided into two categories based on the route of gene transfer: The ex vivo route and the in vivo route.^1^ In the ex vivo route, the gene of interest is introduced into cells extracted from the patient. The cells are then delivered back into the human body after in vitro proliferation of the cells, screening, drug treatment, or a series of other operations.^14^ This approach requires a gene delivery vector, the DNA or RNA that makes up the gene itself, and technologically advanced facilities for processing the cells. For the in vivo route, a genetic material vector is injected directly into patients.^20^ This approach is similar to other types of drug delivery systems. Compared with the ex vivo route, the in vivo approach is more dependent on gene delivery vehicles.

### 2.2 CAR gene therapy

DNA‐based gene therapies involve the delivery of DNA fragments and CAR T‐cell therapy. CAR T‐cell therapy involves the in vitro genetic modification of autologous T‐cells in patients to encode a chimeric receptor that binds to a specific tumor antigen. These cells are then reinfused into the patient.^21^, ^22^ CAR T‐cell therapies have been successful in treating hematologic malignancies.^23^ Autologous T‐cells are genetically modified to express CARs targeting the B‐cell antigen CD19, which has led to remarkable clinical responses in patients with B‐cell‐related malignancies. However, CAR T‐cell therapies have several major limitations, including life‐threatening CAR T cell‐related toxicity, limited efficacy against solid tumors, resistance to B‐cell malignancies, antigen escape, limited persistence, poor transport, tumor infiltration, and an immunosuppressive microenvironment.^22^ Transport can be improved by local injections^24^, ^25^ or chemokine receptors on CAR T‐cells.^26^, ^27^ Immunosuppressive microenvironments can be tackled using immune checkpoint inhibition. Toxicity can be reduced while optimizing efficacy by altering the structure of CARs, modifying CAR‐transduced T cells, or implementing “off‐switch” or suicide gene strategies.^28^, ^29^ Mesothelin is another factor that has been investigated in studies on CAR T‐cell therapies. Mesothelin is a tumor differentiation antigen that is overexpressed in a wide range of solid tumors.^30^ Several mesothelin‐targeted CAR T‐cell therapies have been developed to treat solid tumors,^31^, ^32^ but the use of CAR T‐cell therapies for solid tumors remains challenging because of the biocomplexity of the solid tumor microenvironment.

### 2.3 RNA‐based gene therapy

RNA‐based therapies comprise two categories. The first includes RNA molecules or analogs used directly as therapeutic agents and the second includes RNA‐targeted small‐molecule drugs. Various RNA delivery vectors have been investigated for the widespread use of RNA therapies. Among RNA delivery systems, biomaterials for nonviral RNA delivery have exhibited excellent performance.^33^

ASOs are single‐stranded nucleic acid polymers comprising 18−30 bases that can combine with specific sequences of target mRNAs through complementary base pairing to upregulate or downregulate protein expression.^34^, ^35^ Antisense RNAs comprise a class of small RNAs without coding functions of their own, but can suppress the function of target RNAs and regulate the expression of the corresponding gene by complementarily binding to the target RNA, especially to specific regions of the mRNA, through hydrogen bonds between paired bases.

RNA interference (RNAi) is a sequence‐specific way to knock down the expression of target genes through double‐stranded RNA (dsRNA) mediated mRNA degradation or inhibition of mRNA translation.^36^ RNAi involves posttranscriptional gene silencing, an effect that may be mediated by miRNA and siRNA. In this process, mRNAs with homologous sequences to the dsRNA are degraded, suppressing the expression of the gene of interest. miRNAs are endogenous, highly conserved, and small noncoding RNAs comprising approximately 22 nucleotides. miRNAs regulate the expression of coding genes by incompletely binding to their 3′‐untranslated regions and are further considered key regulators of target gene expression.^37^ Long dsRNA is cleaved by an intracellular Dicer into short dsRNA comprising 20−25 nucleotides. These molecules are siRNA.^38^ Compared with conventional gene therapies, siRNAs can effectively silence diseased genes and knock down their function. Recent US FDA approval of the first and second siRNA drugs has marked the beginning of the era of RNAi therapies. These drugs include Patisiran, which is an siRNA formulated as a lipid complex for delivery to hepatocytes,^39^ and Givosiran, which is an siRNA that is bound to the N‐acetyl‐D‐galactosamine (GalNAc) ligand for the asialoglycoprotein receptor‐mediated targeted delivery to hepatocytes,^40^ respectively.^41^

RNA activation (RNAa) is mediated by small dsRNA, targets gene regulatory sequences such as promoters and various noncoding regions, and involves transcriptional and epigenetic alterations. Small dsRNAs that act as activators of gene expression have been defined as small activating RNAs (saRNAs) or antigene RNAs.^42^ RNAa requires additional steps, such as saRNA crossing the nuclear membrane, with the mechanism being more complex than that of RNAi,^43^ thus RNAa occurs later than RNAi. Furthermore, the effects of saRNA‐induced gene activation last much longer than those of downregulated expression triggered by RNAi. saRNA‐induced gene activation by RNAa has also been implemented as a novel strategy to treat cancer. For instance, p21‐saRNA‐322 impeded the growth of colorectal cancer by activating the p21 gene.^44^

Another RNA‐based therapy involves mRNA, which can encode proteins that exert therapeutic activities. In eukaryotes, RNA polymerase synthesizes precursor mRNA in vivo when it converts genes into primary mRNA transcripts. During mRNA splicing, introns are removed from the transcript, whereas exons are spliced to produce mature mRNA. The advantages of mRNA are as follows: (1) mRNA can theoretically express any protein and thus treating an extensive range of diseases. (2) DNA can be transcribed only into mRNA after entering the nucleus. However, mRNA need not enter the nucleus and can initiate protein translation in the cytoplasm. Therefore, it is more efficient than DNA. (3) mRNA does not affect genetic information by inserting itself into the genome as DNA and viral vectors do. mRNA‐encoded proteins are only expressed momentarily and are quickly degraded afterwards without the risk of gene integration. (4) Compared with proteins and viruses, mRNA can quickly translate all the proteins in cells. Moreover, industrial production is simple and inexpensive. In addition, mRNA chains are long‐chain macromolecules with negative charges, while the surfaces of cell membranes also have negative charges. It is difficult for mRNA molecules to cross the cell membrane and enter the cell because of electrostatic repulsion. Moreover, because mRNA molecules are single‐stranded, they are extremely fragile and can rapidly be degraded by various enzymes in the body. The information encoded by mRNA further covers the sequences of ribosomal proteins and must be delivered to cells to encode proteins. Therefore, there are two barriers to delivering mRNA into cells: The enzymatic degradation of mRNA while being delivered and its electrostatic repulsion with the membrane barrier. Therefore, special modifications or encapsulated delivery systems are necessary to achieve the intracellular expression of mRNA and change its intracellular biodistribution, cellular targeting of mRNA, and uptake mechanisms to facilitate mRNA delivery.^45^

### 2.4 CRISPR/Cas9‐mediated gene therapy

The CRISPR system recognizes and degrades foreign genetic materials, such as RNA and DNA, for bacterial defense by integrating short exogenous sequences into the bacterial genome and transcribing them into CRISPR RNAs (crRNAs).^46^ The CRISPR/Cas9 system, from which the type II CRISPR system was modified, comprises the Cas9 protein and single guide RNA (sgRNA). Under the guidance of sgRNA, Cas9 cleaves the target DNA by recognizing adjacent protospacer motifs. After the target DNA breaks, repair mechanisms are initiated, including the cellular nonhomologous end‐joining repair mechanism, which can rejoin the genomic DNA at the break and introduce insertion or deletion mutations. Another repair mechanism is homologous recombination repair (HDR), which requires the involvement of HDR templates to insert or replace gene fragments at the break.^47^

CRISPR/Cas9 has become a research hotspot and has been used to edit genomes to treat various diseases. CRISPR/Cas9 can be delivered in three ways: (1) the delivery of plasmid DNA encoding Cas9 and sgRNA; (2) the delivery of Cas9 mRNA and sgRNA, during which mRNA is converted to Cas9 nuclease through translation in the cytoplasm; (3) and the delivery of ribonucleoproteins (RNPs), which comprise a complex of Cas9 protein and sgRNA. The latter bypasses the transcription and translation process and provides the fastest form of gene editing.^42^ There are advantages and disadvantages of each delivery method, mainly in terms of efficiency, off‐targeting, and immunotoxicity (Table 1).

**TABLE 1:** Advantages and disadvantages of the CRISPR–Cas9 system.

Delivery format	Advantages	Disadvantages	References
Plasmid‐based delivery method: plasmid DNA of CRISPR–Cas9	Fast (the slowest of the three methods), simple, and low cost Higher stability than protein and mRNA	Difficult to deliver, release and translate into protein, large encoding pDNA (>7 kb) Complex and takes a long time Easy to produce off‐target effect Plasmid DNA may be randomly integrated into the genomic DNA, easily triggering the body's immune response	^46^, ^48^
RNA‐based delivery method: Cas9 mRNA and sgRNA	Starts gene editing faster, reduces the duration of gene editing, and more efficient Reduces off‐target effects	The most unstable (Shorter gene expression time)	^47^, ^49^, ^50^
Protein‐based delivery method: Cas9/sgRNA ribonucleoprotein (RNP)	The fastest genome editing Significantly reduced off‐target effects and toxicity	Cas9 proteins have a considerable molecular weight (160 KDa), which makes it challenging to achieve efficient delivery of Cas9/sgRNA RNPs Large amounts of highly active Cas9 protein are difficult to obtain owing to high cost and bacterial endotoxin contamination Easy to initiate immune response	^47^, ^51^, ^52^

## 3 EXOGENOUS GENE TRANSFER METHODS

Genes are polyanions of repeating chains of phosphate groups. Cell membranes are also negatively charged; thus, genes cannot interact with cell membranes due to electrostatic repulsion. Therefore, internalization and transfection of genes into cells is difficult to achieve. There are three methods available to address this obstacle: (1) physical stimulation; (2) viral transduction; (3) complexation with biological materials.

### 3.1 Physical methods

Physical stimulation has been shown to enhance gene transfer into cells because it allows genes to move near the cell membrane. It also allows for the temporary microdisruption of the cell membrane. Various physical methods have been investigated to enhance gene expression in vivo using needle injections, ballistic pressure injections (gene gun), electric fields (electroporation), hydrodynamic pressure (water perforation), magnetic fields (magnetic transfection method), and ultrasounds (ultrasonic perforation).^53^ In addition, most biomaterial‐based carriers are based on cationic polymers and lipids, which allow genes to maintain physiological pH. These complexes not only protect genes from nuclease attacks, but also promote cellular internalization through electrostatic interactions with cell membranes.

Microinjections comprise a nonendocytic driving technique for the precise delivery of molecules and cells into droplets or for the delivery of genes, molecules, proteins, or viruses into individual cells.^54^, ^55^ This technique allows for the rapid delivery of genes into the nuclei. The advantages of microinjections include precise doses and timing, high transduction efficiency, and low cytotoxicity. However, manual microinjections are labor intensive and time consuming. They also comprise an empirical technique, meaning that the cell survival rate after microinjections is heavily dependent on the operator, which limits the application of this technique for numerous cells in samples.^56^ Sun et al.^57^, ^58^ proposed an autonomous microrobot system comprising a pipette holder and a syringe to achieve a high injection success rate.^59^ Despite advances in automated microinjections, current techniques have limitations in terms of accuracy or injection speed. In contrast to active microinjection techniques with injection cycles greater than 0.1 s, Azarmanesh et al.^55^ developed a passive microinjection technique that relies on pressure‐driven fluid flow and pulsating flow patterns within a high‐throughput droplet microfluidic system to generate continuous droplets while rapidly managing microinjection droplets. This technique reduces the injection period to approximately 3 ms and delivers the droplets with minimal errors.^60^ Passive microinjection technologies can work effectively and in a cost‐efficient manner. They can also deliver cells and particles into droplets accurately and noninvasively.^55^ Chen et al.^61^ administered microinjections of oocytes and early embryos at different stages of the development of their experimental mice by combining a suction head pipette and piezo‐assisted micromanipulator, which significantly improved the cell survival rate after microinjections.

Gene guns are devices that deliver foreign molecules into cells. By wrapping DNA in heavy metal particles, such as gold, tungsten, and titanium, and using mechanical forces to launch these particles into the cell, the target genes can be integrated into the target cells. For more than two decades, gene guns have been used to transfer DNA to various biological species. However, cell damage caused by the air pressure (20−60 bar) used by the gene gun system and particle impingements in target tissues are limitations of gene gun use.^20^

Electroporation changes the permeability of a cell membrane by using short, high‐voltage pulsed electric fields to treat molecules and direct the physical delivery of genes to the target tissue.^62^ In recent years, electroporation has been applied to gene therapies that involve CRISPR/Cas9 gene editing systems to treat various diseases.^63^, ^64^ Cells must use electroporated colorimetric dishes or microfluidic devices to be concentrated in small volumes. The cell density typically used in these methods is approximately 0.5−2 × 10^6^ cells/mL.^65^, ^66^, ^67^ This prevents large‐scale implementation for industrial production. Considering the electrodes, electroporation can be used only close to the skin^68^ or to surgically transfer genes to deeper tissues, such as a beating heart.^20^ Electroporated cells are low in vitality,^38^ and their traumatic and tissue contusions can lead to cell death.^69^

Sonoporation, which uses ultrasounds and microbubbles, comprises the ultrasonic transfection method. This method uses high‐intensity ultrasounds to induce instantaneous holes in cell membranes to allow DNA entry. Ultrasound‐mediated gene transfer, or acoustic perforation, is a minimally invasive, nonviral, and clinically transformable gene therapy. Compared with other physical gene delivery methods, such as electroporation, ultrasound‐mediated gene transfer provides better security and is less invasive. Ultrasound‐targeted microbubble destruction (UTMD) refers to the targeted delivery of drugs or genes using ultrasound and microbubbles. Several studies have shown that UTMD may enhance local gene delivery. Microbubbles have a significant cumulative effect on ultrasonic membrane penetration. The disturbance of the cell membrane and blood vessel walls by ultrasonic oxidation of microbubbles can increase permeability and gap delivery.^70^ Intracellular reactive oxygen species produced by ultrasound may contribute to cell membrane penetration without affecting cell viability.^71^ The main advantage of ultrasound‐mediated microbubble‐assisted transfection is the ability to deliver and express nucleic acid only in sonar regions rather than in nontargeted organs.^72^ Ultrasound‐mediated microbubble cavitation was used by Zhang et al.^73^ to facilitate AAV‐mediated cochlear gene transfection across the round‐window membrane.

**FIGURE 3:**
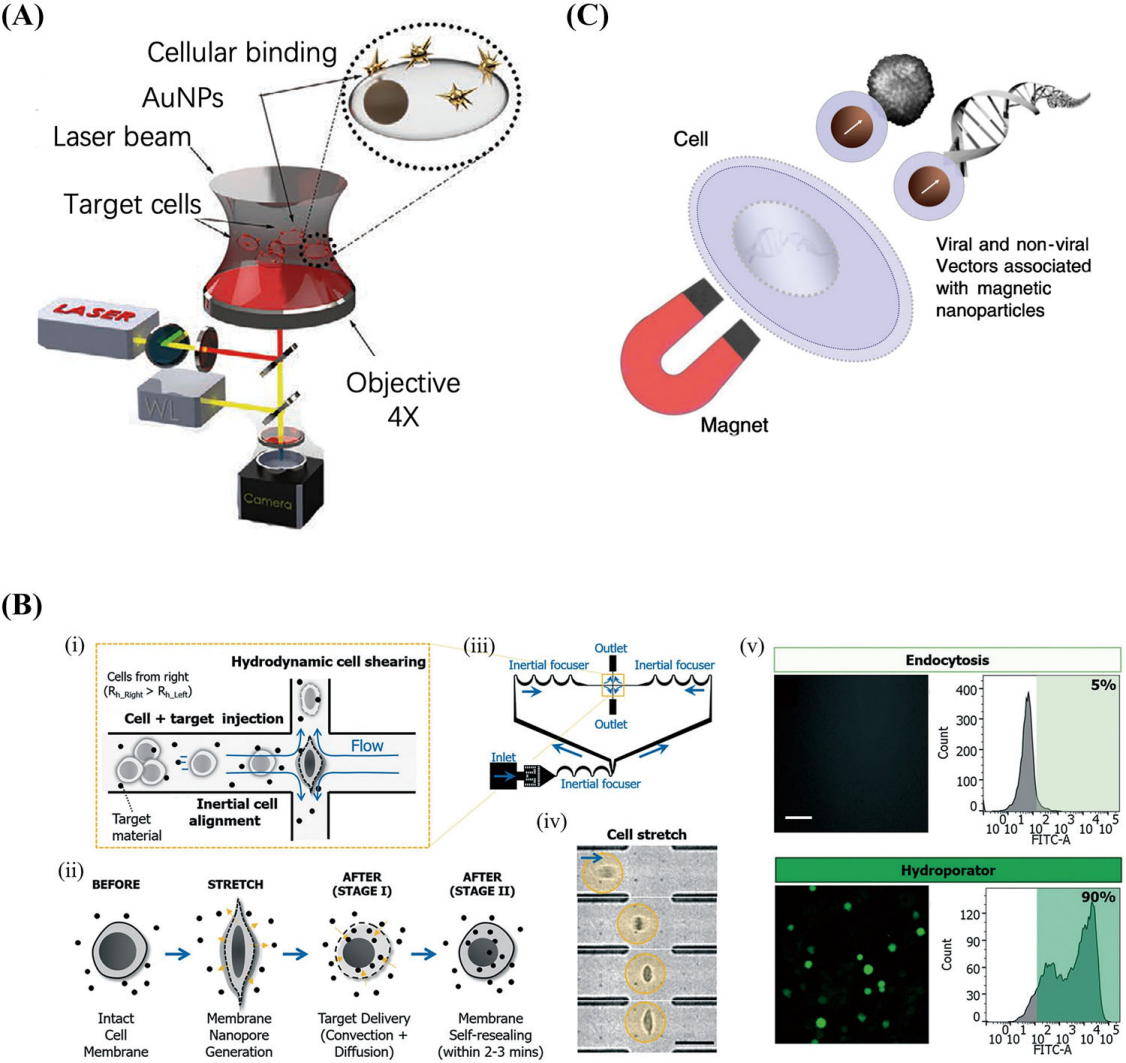
Schematic diagram of the physical methods of exogenous gene therapy. (A) PS laser setup and optical light pathway for irradiation. Reproduced with permission from Ref. 76, Copyright 2021 © Wiley‐VCH GmbH. (B) Hydroporator: hydrodynamic cell deformation‐induced intracellular delivery of nanomaterials. (i) the design and operation principles. (ii) The delivery mechanism (iii) layout of hydroporator (iv) High‐speed microscope images. (v) FITC‐dextran in K562 cells using hydroporator. Reproduced with permission from Ref. 80, Copyright 2019 © Royal Society of Chemistry. (C) Principle of magnetofection. Reproduced with permission from Ref. 81, Copyright 2011 © Elsevier B.V.

Photoporation is the process of delivering nucleic acids mediated by membrane permeabilization induced by high‐intensity and short‐duration laser pulses on individual cells.^74^, ^75^ Hasanzadeh Kafshgari et al.^76^ proposed antibody‐functionalized gold nanostar‐mediated photoporation to efficiently and selectively transfect specific cells with siRNA, mRNA, or Cas9‐RNPs for their targeted delivery to human retinal pigment epithelial (RPE) cells (Figure 3A). In addition, the concept of hydrodynamic transport‐based water perforation was established in 1999 to achieve effective plasmid DNA delivery to the liver by rapid tail vein injections of large amounts of DNA solution into mice.^77^, ^78^ Kizer et al. developed an intracellular delivery platform called a “hydroporator,” which enables the rapid intracellular delivery of high‐throughput carrier‐free nanomaterials and DNA origami nanostructures^79^ using only fluid inertia while maintaining biological stability in living cells (Figure 3B).^80^

Another physical gene transfer method is magnetic transfection, or magnetofection. This method involves linking a viral or nonviral gene delivery vector with magnetic nanoparticles and magnetically directing the vector to the target cells for fast and efficient nucleic acid delivery (Figure 3C).^81^ The main advantages of magnetic transfection include its ability to facilitate fast and efficient transfection at low carrier doses. This also allows remote control of vector targeting in vivo. Plank et al.^82^ combined vectors and magnetic particles through appropriate bonds and salt‐induced colloidal aggregation. If these particles were mixed with DNA, liposomes, or polymeric complexes such as polyethyleneimine (PEI)‐DNA in a salty buffer, they would bind or copolymerize with these compounds. Under a magnetic field, cells were incubated with a vector‐magnetic particle mixture that attracted the particles into the cells.^82^ The magnetic particles used were mainly superparamagnetic iron oxide nanoparticles, which were coated with highly biocompatible polymers, such as PEI,^83^ glucan,^84^ and poly(amidoamine) (PAMAM) dendritic macromolecules.^85^ The results of magnetic transfection comprise the rapid deposition of a full‐dose vehicle on target cells, which further leads to a high percentage of cells being rapidly transfected within minutes.^86^

### 3.2 Viral vectors

Studies have shown that viral vector‐mediated gene delivery is the most efficient method of gene transfer. Viral vectors are therefore the most commonly used gene therapy vectors. The production of nonpathogenic viruses for gene therapy has increased in recent years. These viruses include retroviruses, lentiviruses, adenoviruses, and AAVs.^87^

Retroviruses are RNA viruses composed of two RNA strands packaged with proteins. In addition, they comprise a simple genome structure and an expression cassette that can be replaced by therapeutic genes. When retroviruses enter host cells, the viral RNA is reverse‐transcribed into a double‐stranded DNA molecule, enters the nuclei of the cells, and randomly integrates into the host cell genome as a “provirus.” The provirus is then transcribed into RNA, after which the packaging protein is synthesized. The transcribed RNA genome is then released outside the cell to undergo the cell cycle.^88^

Lentiviral vectors are gene therapy vectors developed based on the human immunodeficiency type I virus (HIV‐1). Lentiviruses comprise a family of retroviruses, but unlike general retroviruses, lentiviruses have a wider host range for infection of both dividing and nondividing cells. Research on lentiviral vectors is moving rapidly and aims to efficiently integrate foreign genes into the genomes of host cells for long‐lasting expression.^89^

Adenoviruses are large molecules with genomes of approximately 36 kb. They are double‐stranded, nonenveloped DNA viruses. Adenoviruses enter cells through receptor‐mediated endocytosis, after which their genomes are transferred to the nucleus, but remain outside the chromosomes without integrating into the host cell genome. The host cell range of adenoviruses is broad and can infect terminally differentiated cells that divide and do not divide, such as neurons.^90^

AAVs are single‐stranded, linear, DNA‐deficient viruses without envelopes. AAVs comprise an icosahedral envelope protein with a diameter of approximately 22 nm. Furthermore, they comprise a single‐stranded linear DNA genome of approximately 4.7 kb. AAVs cannot replicate independently; their replication depends on secondary viruses, such as adenoviruses. AAV vectors comprise a new class of safety vectors that have been widely studied and are not pathogenic to humans.^91^, ^92^

### 3.3 Nonviral vector delivery systems

Nonviral vectors must be able to mimic viral functions, such as expressing nucleic acids, targeted cell attachment and internalization, endosomal escape, and nuclear transfer.^93^ Most gene vectors enter cells primarily through the endocytic pathway. After being internalized through the endocytic pathway, genes are carried to endosomal compartments, where they are then enzymatically degraded after endosomal‐lysosomal fusion. An effective gene vector should promote the rapid escape of genes from endosomes. Numerous studies have assessed materials and methods to promote endosome escape and constructed highly efficient and low toxicity nonviral gene vectors.

Biomaterials include three categories: metallic, inorganic, and organic materials. Organic materials mainly include polymers, which can be categorized as natural polymers, such as chitosan, hyaluronic acid (HA), and cyclodextrin (CD), or synthetic polymers, such as polycationic peptides, PEI, polyesters, polyethylene glycol (PEG), and polyamide (PAA), depending on their source. Natural cationic polymers are often nontoxic, less immunogenic, and biodegradable; thus, many have been approved by the FDA as safe biomaterials. Synthetic polymers have the advantage of being modified by combining bioactive moieties and functional groups, but are limited by their excessive cytotoxic effects. In addition, because of their high positive charge, they can bind to anionic biomolecules in sera, which can lead to off‐target side effects. However, low‐molecular‐weight cationic polymers have good cell tolerance, but unfortunately, they are inefficient in cellular delivery.^94^

Biomaterial‐based vectors often have well‐defined structures, designed functions, no immunogenicity, and the potential for large‐scale production.^95^ Another advantage of these carriers is that they protect their cargo from enzymatic degradation and immune recognition.^96^ Therefore, cationic lipids, polymers, or lipid–polymer hybrids have been developed for gene delivery by being compounded with negatively charged nucleic acids.^16^

## 4 BIOMATERIAL‐BASED NUCLEIC ACID DELIVERY SYSTEMS

According to the composition of biomaterials, biomaterial‐based delivery systems can primarily be classified into liposomes and their derivatives, peptides, polymeric nanoparticles (polyesters, polyethyleneimine, PEG, polyamino acids, and natural polymers), inorganic nanoparticles, dendrimers, extracellular vesicles (EVs), and so on. Here, we highlight the biological characteristics and the physical and chemical properties of these biomaterials, alongside their advantages and disadvantages as gene transfer vehicles.

### 4.1 Liposomes and their derivatives

Liposomes are self‐assembled vesicles composed of phospholipids with polar head and nonpolar tail groups and stabilizers, such as cholesterol. The cationic nature of liposomal systems and their unique ability to capture lipophilic and hydrophilic compounds allow for the encapsulation of nucleic acids and various drugs in these vesicles. Once inside cells, liposomes are processed via the endocytic pathway, after which nucleic acids are released from endosomes or vectors into the cytoplasm. Liposome formulations are characterized by particle sizes, charges, number of sheets, lipid composition, and surface modifications with polymers and ligands, which determine their stability. Over the past two decades, lipid cations have been extensively studied and modified to reduce cytotoxicity and enhance gene expression levels. For example, the binding of PEG polymers to liposomal membranes has been demonstrated to be a critical strategy for elongating the circulation times of carriers and the prevention of the reticuloendothelial system (RES)‐mediated carrier removal through spatial stabilization.^97^

Micelles are thermodynamically stable micellar agglomerates of self‐assembled and ordered molecules in aqueous solutions when the surface activator reaches a certain concentration. Lipid micelles are tiny droplets generated when the hydrophilic head of a phospholipid is exposed to water, whereas the hydrophobic tail clusters to avoid water molecules. Phospholipids with short compact tails often form micelles, whereas those with longer tails form liposomes.^98^ Surfactants are amphiphilic molecules with lipophilic tails that cluster inside micelles due to hydrophobicity and hydrophilic head ends that extend outward because of polarity and protect the hydrophobic groups inside the micelles. Micelles may act as vectors for the core genetic material and they can easily enter tissues while rarely triggering immune responses.^99^

Lipid nanoparticles (LNPs) are lipid vesicles with a homogeneous lipid core.^100^ LNPs include ionizable and cationic lipids, cholesterol, phospholipids, and PEG lipids, of which ionizable lipids play an important role in protecting nucleic acids from nuclease‐mediated degradation. The encapsulated drugs are distributed within the hydrophobic layer, hydrophilic cavity, or on the surface of the nanoparticles. Moreover, the external surface of LNPs can be coupled to targeting ligands. Thus, the surface chemistry of LNPs is highly programmable.^101^ Lipid polyplexes are double‐layered structures with polymer‐encapsulated mRNA as the inner core and encapsulated phospholipids as the outer shell. Lipid polyplexes provide better encapsulation and protection for mRNA than conventional LNPs and allow gradual release of mRNA molecules as the polymer degrades.^102^ Lipid polyplexes (Lipo‐polyplexes) have a balance between stability and cargo release that improves the efficiency of transfection while reducing cytotoxicity.^103^, ^104^

### 4.2 Peptides

Peptides can be integrated as functional components in nonviral gene delivery systems to overcome biological barriers.^97^ Functional peptides that can be incorporated to improve the low transfection efficiency of nonviral systems and ultimately enhance gene expression can be classified according to their use in overcoming biological barriers. These classifications include (1) cell‐penetrating peptides (CPPs), which facilitate cell entry; (2) targeting peptides, which improve specific cell binding; (3) endosomal disruption peptides, which overcome endosomal trap barriers; and (4) nuclear localization signal peptides, which facilitate efficient nuclear entry.

CPPs are short peptides of only 30 amino acids. They can penetrate biological membranes and deliver various bioactive substances into cells. CPPs are positively charged alkaline peptides at physiological pH and have large cargo loads, high cellular permeability, and can cross the membranes of different cell types while exhibiting low cytotoxicity and causing no immune response.^105^, ^106^ In addition, they have been used as carriers for siRNA, plasmid DNA, small molecules, proteins, and other peptides in vitro and in vivo. Peptides can self‐assemble with nucleic acids to form peptide‐based nanoparticles (PBNs), thus opening peptides to the field of nanomedicine.^107^ Depending on their origin, CPPs can be subdivided into three categories: protein‐derived peptides, chimeric peptides, and synthetic peptides.^108^ CPPs can also be classified into the following types according to their physicochemical properties: Cationic peptides, such as transactivator (TAT) and poly(alpha‐l‐lysine) (PLL), which comprise short sequences of amino acids such as arginine, lysine, and histidine; amphiphilic peptides, such as MPG (GLAFLGFLGAAGSTMGAWSQPKKKRKV), which are peptides with polar and nonpolar structural domains; and hydrophobic peptides, which are formed from nonpolar residues of valine, leucine, and tryptophan.^109^

Protein‐derived CPPs comprise polypeptide regions of naturally occurring proteins that primarily translocate proteins into cells. The TAT sequence (RKKRRQRRR) originates from the TAT transcription factor of HIV. Bahadoran et al.^110^ developed PAMAM dendrimers coupled to TAT. When TAT/PAMAM nanoparticles were formulated, skin penetration and cellular uptake of plasmid DNA were slightly improved. TAT‐conjugated PAMAM achieved transdermal delivery of DNA better than unmodified PAMAM dendrimer.^110^ Furthermore, CPP‐ and cationic poly(ethyleneimine)‐conjugated gold nanoparticles (AuNPs) were developed by Niu et al.^111^ for transdermal delivery of plasmid DNA to reverse the progression and metastasis of cutaneous melanoma. Synthetic CPPs are synthetic poly‐arginine‐ or poly‐lysine‐based CPPs or more efficient membrane‐penetrating CPPs modified from natural CPPs, such as MPG and Pep‐1 (KETWWETWWTEWSQPKKRKV). Pep‐1 was the first approved CPP on the market, with the trade name Chariot. Peptides made from lysine and arginine only, called “poly‐Lys” and “poly‐Arg,” respectively, were the first to be evaluated as artificial CPPs due to their ability to be internalized in living cells. When used as carriers, poly‐Lys PLLs are usually bound to other proteins and synthetic compounds, such as PEI, whereas unbound poly‐Lys has a lower transfection rate.^112^ Chimeric CPPs are synthetic peptides formed by the fusion of two or more naturally occurring sequences. Amphipathic CPPs derived through covalently linking hydrophobic structural domains to effectively target the nuclear localization sequences (NLS) of cell membranes are also chimeric peptides. For example, MPG and Pep‐1 based on simian virus 40 NLS PKKRKV.^109^

### 4.3 Polymeric nanoparticles

Nanoparticles are less than 100 nm in diameter and vary in shape, size, and materials used to prepare them. Nanomedicines are often intended to enhance therapeutic efficacy by efficiently delivering drugs to the target site and/or reducing toxicity by minimizing their accumulation in healthy body sites. Nanoencapsulation can protect therapeutic agents from degradation in the biological environment and can further provide solubilization.^113^ Nanoparticles have positively charged surfaces, whereas cell surfaces are negatively charged; therefore, nanoparticles can enter target cells efficiently. Once inside the cell, nanoparticles undergo lysosomal escape and disassemble within the cytoplasm, releasing nucleic acids that enter the nucleus through nuclear pores and rely on the host enzyme system to express functional proteins for therapeutic purposes. As nanoparticles enter the circulation of the host, they encounter enzymes, plasma proteins, RES, phagocytes, and other components. The protein layer that forms on their surface is called a “protein crown.” Nanoparticles with protein crowns are readily recognized by the immune system, which leads to rapid clearance by the mononuclear phagocyte system (MPS). Nanoparticles with protein crowns also accumulate in the small pulmonary capillaries and cause serious toxic effects.^114^ The prolonged circulation of nanoparticles in the blood is a prerequisite for achieving the controlled and passively or actively targeted release of the encapsulated gene or drug at the desired site of action. The most widely used method for masking or camouflaging nanoparticles involves the adsorption, grafting, or binding of PEG or other hydrophilic polymers, such as polysaccharides, to the surfaces of the particles, reducing toxic side effects and prolongs the circulation of the particles in the blood. The main factors that affect the circulation of nanoparticles are the particle size, surface charge, and hydrophilicity. The surface modifications of polymeric nanoparticles involve PEGs and polysaccharides.^115^ In this section, we focus on synthetic and natural polymeric nanoparticles.

#### 4.3.1 Polyesters

For decades, polyesters such as poly(lactic acid) (PLA), poly(lactic acid‐glycolic acid) (PLGA), poly(ε‐carprolactone) (PCL), and poly(β‐amino ester) (PBAE) have been clinically used as biomaterials for the manufacture of medical devices. PLA, PLGA, PCL, and PBAE are not only degradable in the physiological environment but also bioresorbable. To make polyesters positively charged, small cationic molecules can be introduced as side groups through chemical modifications. Cationic polymers, such as PEI, PLL, and chitosan, have been conjugated to the anionic surfaces of PLA and PLGA nanoparticles to form cationic surfaces for the uptake of nucleic acids.^116^ In addition, a PEG block is typically included to protect the positive charges and minimize nonspecific interactions with serum proteins.^117^

PLGA is a randomly polymerized amorphous copolymer of lactic acid and hydroxy acetic acid. It is biodegradable, biocompatible, and has good physical ductility for capsule and film formation. PLGA polymers have been approved by the US FDA for therapeutic applications since 1969.^118^ CRISPR/Cas9 plasmids of approximately 8500 bp are susceptible to shear deterioration because of their high molecular weights. Therefore, Jo et al.^119^ explored the engineering and processing steps required to fabricate fluorescently labeled PEG–PLGA nanoparticles for the encapsulation of high‐molecular‐weight plasmid DNA. PLA may be obtained by polymerizing lactic acid produced by biofermentation. In addition, to block copolymers based on conventional aliphatic polyesters, alternative copolymers such as PBAE, comprising ester and cationic units, have been developed for the delivery of nucleic acids.^117^

PBAEs can be prepared by stepwise growth polymerization or ring opening polymerization (ROP).^120^ PBAEs can compress nucleic acids into nanoscale particles, and the strength of the binding of PBAEs to nucleic acids can be adjusted by using the different end caps on the PBAE structure.^121^, ^122^ When there is a larger number of amine‐containing end caps, nucleic acid loads are compressed to form smaller nanoparticles,^123^ which may then be taken up by cells. The increased amount of tertiary amines provided by the branched PBAE structure can lead to greater protonation in low‐pH buffers, which results in the improved condensation of nucleic acids. Larger numbers of end groups can also lead to greater chemical flexibility and a stronger effect on transfection.^124^, ^125^ Although PBAEs are cationic, their charge density is lower than other polymers traditionally used for gene delivery, such as PEI and PLL. Therefore, PBAEs must be used in higher quantities to achieve the same degree of nucleic acid binding as other polymers. Sunshine et al.^126^ found that a larger mass of PBAE was required to achieve DNA binding and buffering capacities comparable to those caused by PEI; however, the PBAEs in this study allowed for greater transfection than PEI due to the lower toxicity of the PBAEs tested. The hydrolytic degradation of the ester bond of PBAE has been shown to result in relatively low toxicity, allowing higher amounts of polymers to be used in nanoparticle formulations without concerns of toxicity. To address the drawback of relatively slow and uncontrolled nucleic acid release, researchers have chemically manipulated PBAEs (e.g., through the partial introduction of photosensitive 2‐nitrobenzene to the backbone or binding of disulfide bonds), which has resulted in the rapid degradation of nanoparticles in response to stimuli or environmental changes to further facilitate the rapid and triggered release of nucleic acid cargoes within cells.^127^

The cationic nature of PBAE may pose challenges. Considering that the formation of nanoparticles is primarily driven by electrostatic interactions, PBAE is typically limited to the delivery of anionic cargoes. Although this is well suited for the delivery of highly negatively charged nucleic acids, it may limit applications that require the codelivery of another cargo. To bypass the obstacles involved in using Cas9‐RNPs as a gene delivery carrier, Rui et al.^128^ developed carboxylate‐terminated PBAEs that can coencapsulate anionic sgRNA through electrostatic interactions and Cas9‐RNPs through hydrogen bonding and hydrophobic effects. Excessive positive charges can also lead to cytotoxicity by disrupting the cell membrane. This can be addressed by mixing PBAE with PLGA and coating the surfaces of PBAE/PLGA/DNA nanoparticles with CPPs.^129^ This results in the overall surface charge becoming less positive and improves the safety of PBAE as a vector. Nanoparticles can also be modified using PEG to reduce their surface charges and improve mobility. Cationic PBAE nanoparticles have been found to exhibit immunomodulatory properties. Andorko et al.^130^ reported that although free PBAE in solution is immunologically inert, nanoparticles formed by PBAE complexes with polyanions can lead to the activation of antigen‐presenting cells. For gene vaccines or therapies that require immune activation, these properties of PBAE may be advantageous. PBAE can be used not only as a core material for nanoparticles, but also as a coating for other gene delivery vectors. This strategy uses PBAEs to overcome gene delivery barriers such as cellular internalization and endosomal escape, whereas other core materials can provide stability, the ability to treat diseased cells, or enhance nucleic acid loading. PBAEs have also been used to encapsulate organic delivery vectors, such as *Escherichia coli* (*E. coli*), to improve gene delivery. PBAE‐based hybrid materials have also shown great promise in facilitating effective gene delivery, as they allow more freedom in their design and use the advantages of different materials to achieve desired therapeutic effects.^127^

#### 4.3.2 Polyethyleneimine

PEI is a large organic molecule with a high cationic charge density. In its structure, the combinations of two neighboring carbon atoms or three neighboring atoms in the monomer (─CH2─CH2─NH─) comprise protonatable amino nitrogen atoms, which make the polymer network an effective “proton sponge” at almost any pH value. This allows PEI to facilitate gene transfer.^131^, ^132^, ^133^ PEI binds to negatively charged nucleic acid molecules to form a positively charged complex that interacts with cell membrane surface anions and readily enters the cell through endocytosis. Following endocytosis, the target gene or PEI complex leaves the endosomal compartment and, due to the proton buffering capacity of PEI, causes the collapse and rupture of the intracellular endosome. This results in endosomal escape and prevents the degradation of the target gene within the lysosome.^134^, ^135^ The complex must then reach the nucleus. PEI uses its charged nitrogen atoms to extend the transport time of the endosome. Because the nitrogen atoms in the PEI structure are close together, numerous amines are not protonated at physiological pH. This makes PEI a good buffer, or proton sponge, as it delays acidification and fusion with the lysosome, protecting DNA from degradation.^131^

PEI has a high transfection rate, but can induce cytotoxicity due to cell membrane instability and/or interference with host gene expression in the nucleus.^136^, ^137^ Depending on the molecular weight of the polymer, high‐molecular‐weight PEIs (>25 kDa) are more efficient for gene transfer than lower‐molecular‐weight PEIs (<2 kDa), but high‐molecular‐weight PEIs are more cytotoxic and nondegradable, which limits their clinical use.^138^ Low‐molecular‐weight PEIs have low transfection efficiencies due to their insufficient nucleic acid concentrations.^139^ PEI is the most important and commonly used cationic polymer and can be broadly classified into two types: Linear PEI (lPEI) and branched PEI (bPEI). lPEI has a secondary amino group in its backbone and a tertiary amino group at the end of the chain, whereas bPEI contains primary, secondary, and tertiary amines in a ratio of approximately 1:2:1. This endows bPEI with complexing and buffering capacities better than those of lPEI. The loading capacity and transfection efficiency of the target gene fragments of bPEI with similar molecular weights are better than those of lPEI, which makes bPEI more suitable for nonviral gene vector applications. bPEI has a larger number of primary amine groups and allows stable gene vector complexes to be formed. The numbers of secondary and tertiary amine groups in the polymer are correlated with the encapsulation rate of exogenous genes, which may cause higher transfection rates.

The introduction of biodegradable chemical bonds by cross‐linking or the side chain modifications of polymeric carriers can affect the degradation and elimination of the carriers in vivo, which further affects the release of target genes and the cytotoxic effects of the carriers. Acetylation modifications of the polymeric primary and secondary amines have been shown to enhance the release of target gene fragments from the cytoplasm by reducing the cytoplasmic buffering capacity and stability of the polymer.^131^ PEI can be functionalized using various hydrophobic molecules. The introduction of disulfide, imine, or ester bonds can further allow the formation of low‐molecular‐weight PEI hybrids or derivatives. Such derivatives have more biodegradable chemical bonds and can therefore be degraded into low toxicity or nontoxic low‐molecular‐weight PEIs in the body.

#### 4.3.3 Polyethylene glycol

To prevent the recognition and elimination of carriers by MPS, gene vector complexes can be modified with PEG or PEG‐like polymers.^140^ Shielding cationic nonviral carriers by PEGylation creates a hydrate cloud around the nanoparticles, which is the most widely used US FDA‐approved “cloaking” strategy. With this hydrophilic corona, the pharmacokinetic properties and biodistribution of the polymeric carriers can be improved. The extent of this improvement depends on several key factors of PEGylation, including the length, density, and conformation of the PEG chain. Typically, the molecular weight of the PEG must be at least 2 kDa and the surface density should be sufficient to maintain a “brush”‐shaped layer to protect nanoparticles from absorption by serum proteins and the RES.^141^

In addition to polysaccharides, PEG is widely used for the modification of PEIs. Various structural modifications of PEI have been developed to improve the transfection efficiency of polymers. PEGylation decreases the positive charge on the surface of the nanocomplex and reduces the interaction of the complex with plasma proteins and red blood cells. Hydrophilic PEGs have been reported to significantly reduce liposome uptake by hepatocytes and splenic macrophages. This provides a rationale for the use of PEGs to increase the duration of the circulation of PEI complexes.^142^ However, PEGs may also reduce uptake in targeted cells. The introduction of pH‐responsive linkages between nanoparticles and hydrophilic shielding polymers is one technique that may circumvent this problem. Once the nanoparticles enter the slightly acidic conditions of the extracellular matrix, the hydrophilic shielding polymer is shed from the surface of the nanoparticles, exposing the original cationic charge.^114^

#### 4.3.4 Polyamino acids

PLLs comprise a class of water‐soluble cationic biopolymers comprising l‐lysine structural units^143^ and have traditionally been synthesized using three polymerization methods: solid phase peptide synthesis (SPPS), ROP, and chemoenzymatic synthesis.^143^ Considering the protonation of its primary amino group, PLL is positively charged under physiological conditions. PLL has been widely used as a functional biological material for nanocarriers and coating materials. However, interactions between cationic PLL and anionic membranes of erythrocytes and vascular endothelial cells often cause hemolysis and cytotoxicity.^144^ The PLL itself further exhibits moderate to high toxicity. To address this issue, the structure of PLL has been modified with PEG to prepare block copolymers that reduce the cytotoxicity of PLL and mitigate the nonspecific binding of PLL to serum proteins.^145^, ^146^ In addition, various studies have found that the PEGylation of PLL allows the construction of complex micelles with core–shell structures in which plasmid DNA molecules are packaged into a nucleus surrounded by a PEG shell. The spatial exclusion of the PEG shell in these studies allowed for shielding against nucleases and extended the circulation times of the materials in vivo. Both the transfection efficiency and cytotoxicity of poly‐Arg are related to its molecular mass, with higher relative molecular weights resulting in higher gene transfection efficiencies, but also increased cytotoxicity. Poly‐Arg with a low relative molecular mass can be used directly as a gene vector.^147^ In addition, poly‐Args formed by d‐type^148^ and l‐type^149^ arginines have more positive charges distributed inside the molecules than poly‐Args in single configurations. This allows for reduced cytotoxicity. Therefore, these materials are biocompatible and biodegradable, and their disadvantages of low gene transfection efficiency and high cytotoxicity may be mitigated. Poly‐Lys or poly‐Arg peptides are now mainly used as graft sequences on other nanoparticles to increase their cell internalization, so it compares with TAT CPPs.^150^, ^151^

#### 4.3.5 Natural polymers

Polysaccharides, such as chitosan, CD, HA, and dextran, are widely used by researchers for drug and gene delivery methods due to their biocompatibility, biodegradability, low toxicity, and ease of modification.^152^ Chitosan is a linear glucosamine cation polysaccharide. Chitosan has a low cytotoxicity, allows mucosal adhesion, and is biocompatible and biodegradable.^153^ It also has polycationic properties. Given that nucleic acids, cell membranes, and nuclear membranes are negatively charged, chitosan can interact with them in an electrostatic manner, leading to condensation and protection of nucleic acids, uptake of complexes, and nuclear migration.^154^ In addition, the amino group of chitosan can contribute to the endosomal escape of complexes, as it can trigger a proton sponge effect like PEI.^155^, ^156^ The proton sponge effect of the carrier can be improved by chemically modifying the polymer.^157^, ^158^ However, the cationic nature of cationic delivery carriers often results in cytotoxicity when there is no optimal formulation.^159^ Chitosan has been used in gene delivery systems for wound healing due to its inherent antimicrobial activity and its ability to stop bleeding and accelerate wound healing.^154^, ^160^, ^161^ Li et al.^162^ used chitosan‐based nanoparticles delivering both doxorubicin (DOX) and Survivin CRISPR/Cas9‐expressing plasmid or Survivin shRNA‐expressing plasmid to enhance antitumor effects. Chitosan derivatives are used in the development of medical materials and biomedicine. With the development of nanotechnology, chitosan derivatives have been prepared into nanomaterials.^163^ There are numerous methods to prepare chitosan‐based microspheres, including anionic cross‐linking, precipitation, composite coalescence, modified emulsification, ion gels, precipitation‐chemical cross‐linking, glutaraldehyde cross‐linking, thermal cross‐linking, and the ball‐dropping addition method.^164^

HA is a biodegradable, biocompatible, and nonimmunogenic anionic polysaccharide with repeating units of d‐glucuronide and N‐acetyl‐d‐glucosamine. HA typically acts as a hydrophilic shield.^165^, ^166^ PAMAMs have positively charged surfaces, and their toxicity profiles are major issues that limit their clinical use. Surface modifications and/or cationic containment strategies are highly desirable to reduce the toxicity of PAMAMs. Chen et al.^167^ mitigated the risks of positive surface charges of PAMAM dendrimers by coupling HA‐SeSe‐COOH with the cationic siR‐93C@PAMAM.

CDs have been used for numerous years to overcome drug delivery barriers. CDs comprise a family of cyclic oligosaccharides that comprise six, seven, or eight linked glucose units called α‐, β‐, and γ‐CDs, respectively.^168^, ^169^, ^170^, ^171^ Many of the hydroxyl groups found in CDs can be used as reaction sites and replaced with alternative functional groups.^172^ Hydroxyl groups can also be used for bioconjugation or polymerization.^168^ CD and its derivatives are naturally available, water‐soluble, biocompatible, and exert a negligible level of toxicity.^173^ Although “pure” CD vectors exist for gene therapy, CDs are often used as one of several components of gene delivery systems. Supramolecular self‐assembly devices based on CDs and their derivatives are often constructed as gene delivery vehicles, and the addition of CDs can confer many beneficial physicochemical properties throughout the system, such as carrying small‐molecule drugs, acting as connectors or modular components, reducing immunogenicity, and disrupting membranes.^168^ The modularity of CDs has been well studied. For example, it has been evaluated through the synthesis of a cationic B‐CD‐modified PEI, which allowed the termination of the branched arms of PEI and thus reduced its biotoxicity.^174^ The self‐assembling properties of CDs can also be used as a basis for developing protective coatings for complexes or linking polymers, such as PEGs, to form external shields.^175^

### 4.4 Inorganic nanoparticles

**FIGURE 4:**
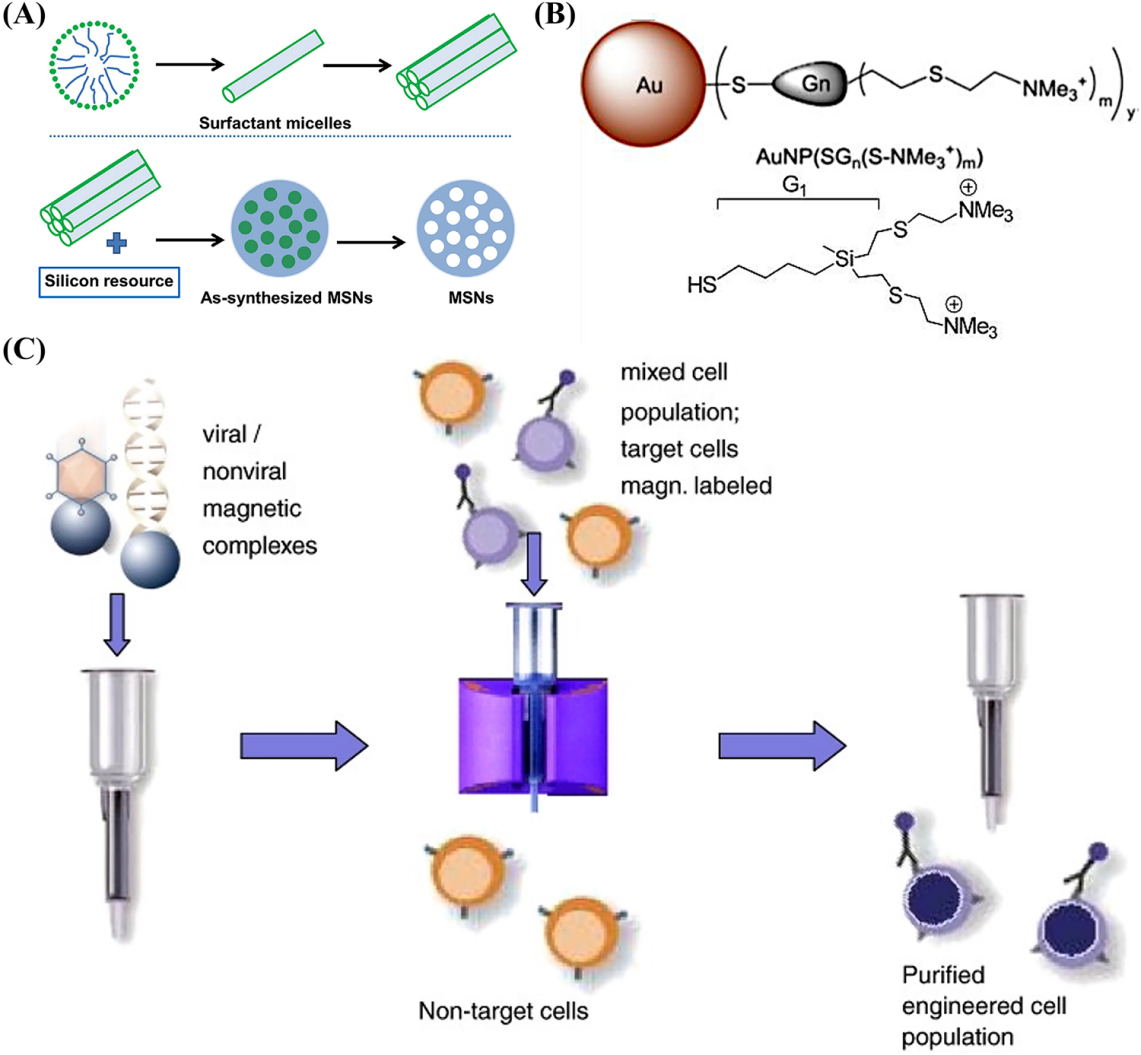
(A) Mesoporous silica nanoparticles (MSNs).^176^ (B) Structures of AuNPs.^181^ (C) Schematic illustration of the magselectofection procedure. Reproduced with permission from Ref. 81, Copyright 2011 © Elsevier B.V.

Inorganic nanoparticles typically have smaller particle sizes, narrower size distributions, and surface chemistry that is more suitable for ligand coupling than that of other polymers or LNPs. The most common inorganic nanoparticles are MSNs, which have custom‐made homogeneous mesoporous structures, high specific surface areas and pore capacities, selective surface functionality, allow control over morphology, and are biocompatible and biodegradable (Figure 4A). MSNs also have high loading capacities and controlled release properties for therapeutic agents if modified with stimulus‐responsive groups, polymers, or proteins.^176^ The mesoporous structures of MSNs allow siRNA to be delivered along with other biomolecules, which results in efficient delivery to target tissues and enhanced gene expression. Mora‐Raimundo et al.^177^ proposed a modified MSN system capable of transporting and delivering the SOST gene, which encodes sclerostin, using siRNA and osteostatin through subcutaneous injections.

Metal nanoparticles, particularly AuNPs, are essential for various biomedical applications^178^ as they can be designed in different sizes and shapes. Metal nanoparticles are highly stable, nontoxic, biocompatible, remarkably reactive, and have a large surface area. In addition, they are electrostatically charged and therefore can be functionalized by other biomolecules, such as nucleic acids and drugs.^179^ Metal nanoparticles can also protect DNA from degraded by nucleases and transfer various drug molecules, nucleic acids, or vaccines to controlled release target sites to treat intracellular diseases.^180^ The functionalization of nanoparticles is significant for reducing their toxic effects, improving their delivery efficiency and accumulation in target tissues, and increasing their stability. More efficient and safer gene carriers based on functionalized metal nanoparticles are being explored. For example, cationic carbo‐silane dendrimer‐modified AuNPs have been synthesized by Abashkin et al.^181^ for the delivery of anticancer siRNAs siBCL‐xL and siMCL‐1 (Figure 4B). Silver NPs are valuable alternatives to AuNPs due to their lower prices and higher reactivity, which increase their range of applications for surface functionalization. Pedziwiatr‐Werbicka et al.^182^ demonstrated that carbo‐silane dendritic surface‐modified silver nanoparticles may be used as carriers for anticancer siRNA, such as siBcl‐xl, and protect the siRNA from enzymatic degradation to ensure effective cellular uptake.

Magnetic nanoparticles are another form of inorganic nanoparticle. Their magnetism allows enrichment, separation, and directional movement or localization (Figure 4C). Magnetic nanoparticles are safe, have a strong binding capacity, low immunogenicity, can protect DNA, exhibit superparamagnetism, and can bind to large DNA fragments. Iron oxide magnetic nanoparticles, such as magnetite Fe_3_O_4_ or its oxidized and more stable forms of magnetite γ‐Fe_2_O_3_, are superior to other metal oxide nanoparticles in terms of biocompatibility and stability. They are also the most commonly used magnetic nanoparticles in biomedical applications. The versatility of superparamagnetic magnetic nanoparticles is attributed to their ability to respond to external magnetic fields and function simultaneously and/or differently with bioactive agents.^183^

### 4.5 Dendrimers

Dendrimers are monodisperse polymers with highly dendritic structures. They comprise an oligomer that is repeatedly and linearly connected by dendrimer units, an inner core, a polymer backbone, and side chains of dendrimer units. Dendritic macromolecules form a dendritic‐like structure by repeatedly growing and branching. As the number of polymerized generations increases, the degree of branching expands, and a closed 3D spherical structure is eventually formed. The spatial steric hindrance of the dendritic units as well as the configuration and flexibility of the dendrimers themselves may be regulated by controlling the structures of the dendritic units, the number of generations, and the distance between the polymer backbones. Dendrimers comprise a new type of polymeric nanostructure and are characterized by hyper‐branched 3D structures with multiple functional groups on their surfaces, which enhance their activity and make them versatile and biocompatible. Their unique properties, such as nanoscale uniform size, high degree of branching, multivalency, water solubility, available internal cavities, and convenient synthesis methods, make them promising reagents for biological and drug delivery applications.^184^

More than 200 dendrimers have been synthesized by the scientific community. These include polypropyleneimine (PPI), PAMAM dendrimers, Fréchet‐type dendrimers, core–shell tecto dendrimers, chiral dendrimers, liquid crystalline dendrimers, peptide dendrimers, multiantigen peptide dendrimers, sugar dendrimers, hybrid dendrimers, and polyester dendrimers. Among these, the most commonly used dendrimers are PAMAMs, PLLs, PEI, and PPI.^184^, ^185^ The surface and internal modifications of PAMAM dendrimers improve their physicochemical properties, cell specificity, and transfection efficiency and can further reduce their cytotoxicity to healthy cells. The three most commonly used modification strategies for PAMAMs include (1) surface modifications with functional groups, (2) hybrid vector formation, and (3) the generation of supramolecular self‐assemblies.^186^ PPI was the first dendrimer introduced and comprises polyalkylamines with primary amine terminal groups and internally composed of several tertiary amine groups. The ability of PPI dendrimers to bind to DNA is enhanced as the number of generations increases, but their toxicity also increases.^187^ Many scholars have chemically modified PPI to improve its performance as a gene vector. For example, Liu et al.^188^ used facile fluorination to prepare efficient and low cytotoxicity gene vectors based on PPI dendrimers.

### 4.6 Extracellular vesicles

**FIGURE 5:**
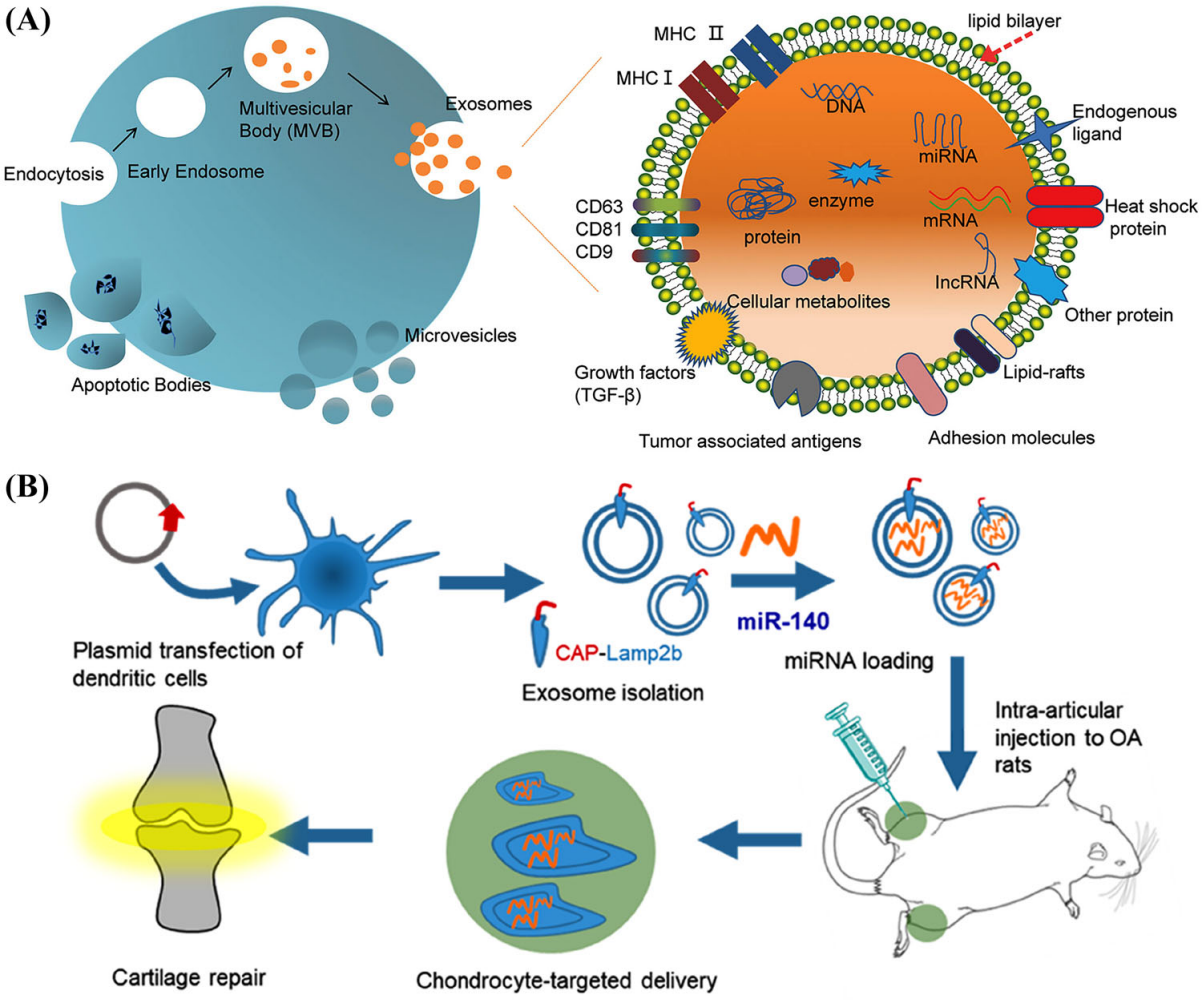
(A) Biogenesis scheme of three kinds of extracellular vesicles (microvesicles, exosomes, and exosome apoptotic bodies) and constituents.^286^ (B) Schematic diagram of the exosome‐based chondrocyte‐targeted miRNA delivery system for the targeted delivery of miR‐140 to treat osteoarthritis. Reproduced with permission from Ref. 195, Copyright 2020 © American Chemical Society.

EVs are vesicle‐like bodies with a double‐layered membrane structure, ranging from 40 to 1000 nm in diameter, and are detached from the cell membrane or secreted by the cell. EVs are defined by the International Society for EVs as lipid vesicles secreted by cells outside the cell. According to the process of their generation, release pathways, sizes, contents, and functions, EVs can be classified into three types: MVs, exosomes, and apoptotic bodies^189^, ^190^ (Figure 5A).

EVs have several advantages as gene delivery systems. First, EVs are multifunctional vectors, so can encapsulate and deliver various biological cargoes. EVs can also naturally cross biological barriers and are able to migrate to tissues or regions without a blood supply. Furthermore, after reaching the target tissue, EVs can remain for a long time. Their low clearance rate can be attributed to their biocompatibility. Another important feature of EVs is that they are genetically modifiable. Their surface proteins can be modified for various purposes. For example, cell‐ or tissue‐targeting peptides can be attached to the surfaces of EVs to reduce systemic toxicity. Several studies have also shown that EV‐based targeted gene therapies can improve therapeutic efficacy and safety.^191^ Kamerkar et al. demonstrated that, unlike liposomes and other synthetic drug nanoparticle vectors, exosomes exhibit an enhanced ability to deliver RNAi and suppress tumor growth. Exosomes contain plasma membrane‐like phospholipids and transmembrane and membrane‐anchored proteins that enhance endocytosis, facilitating the delivery of the internal contents of the vesicles^192^ and reducing their clearance in the circulation. CD47, which is a widely expressed integrin‐associated transmembrane protein in exosomal proteins, protects cells from being removed by monocytes.^193^ The use of exosomes also minimizes the cytotoxic effects observed when synthetic nanoparticles are used in vivo.^194^

Therefore, researchers have been developing exosome‐based delivery systems for gene therapy. For example, Liang et al.^195^ designed an exosome‐based chondrocyte‐targeted miRNA delivery system for the repair of cartilage defects (Figure 5B). Furthermore, Yu et al.^196^ developed a liquid natural extracellular matrix enriched with exosomal miR‐29 to treat pulmonary fibrosis. Exosomes can also serve as vectors for the delivery of CRISPR/Cas9 plasmids. The capacity of natural exosomes to load plasmids is low; therefore, exosomes must be modified to deliver plasmids. Liposomes can package and deliver large plasmids, but display relatively high levels of cytotoxicity due to the unnatural properties of lipids. Exosome–liposome hybrids may be formed by fusion of the lipid bilayer of the exosomal membrane lipid bilayer with the liposome to allow the encapsulation and delivery of large DNA molecules, such as CRISPR/Cas9 expression vectors, and mitigate liposome‐related toxicity. Exosome–liposome hybridization can therefore expand the applications of exosomes in drug delivery. Wan et al.^197^ developed an exosome‐based nanoplatform that allows RNP‐based CRISPR/Cas9 genome editing therapies for liver disease. RNPs can be efficiently loaded into human hepatic stellate cell (LX‐2) derived exosomes by electroporation, and exosomes loaded with Cas9‐RNPs, or exosome^RNP^, can be specifically delivered to the liver. Exosome^RNP^ has been shown to target the p53 upregulated modulator of apoptosis, cyclin E1, and K(lysine) acetyltransferase 5 in gene therapies for mouse models of acute liver injury, chronic liver fibrosis, and hepatocellular carcinoma (HCC), respectively.^197^

## 5 FURTHER DELIVERY STRATEGIES

### 5.1 Microneedles

**FIGURE 6:**
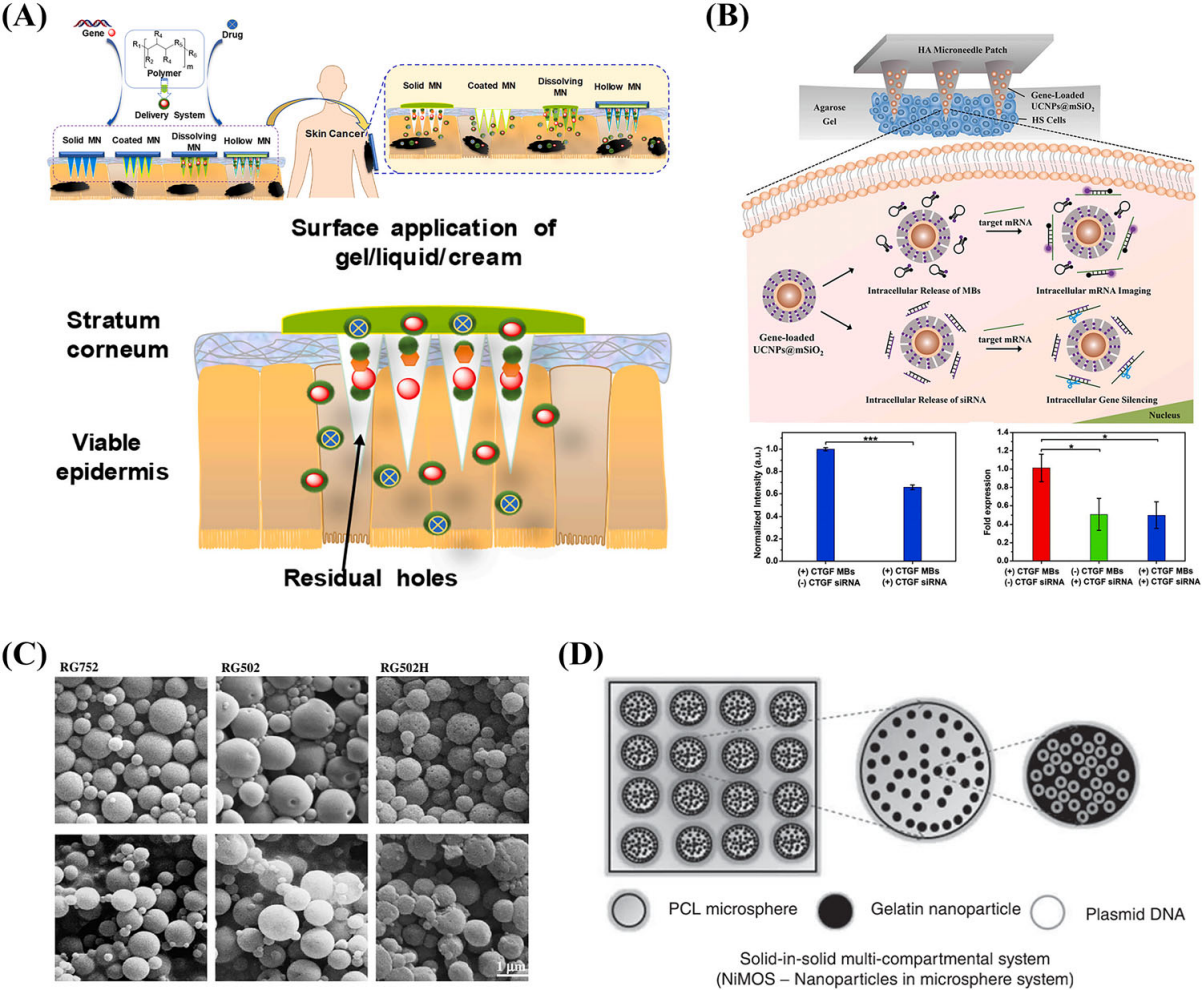
(A) Schematic representation of MN‐mediated gene and drug delivery in the treatment of skin cancer. Reproduced with permission from Ref. 287, Copyright 2021 © Elsevier B.V. (B) Schematic diagram of the nanoparticle‐encapsulated MN system in 3D gels. Reproduced with permission from Ref. 287, Copyright 2021 © Elsevier B.V. (C) SEM of PLGA microparticles with RG752, RG502, and RG502H. Reproduced with permission from Ref. 203, Copyright 2006 © Elsevier B.V. (D) Microsphere‐based multicompartment system for nucleic acid delivery. Reproduced with permission from Ref. 204, Copyright 2017 © Wiley Periodicals, Inc.

Microneedles are micron‐sized (less than 1000 μm), conical, radial, or multifaceted puncturing protrusions that can be solid, hollow, coated, or dissolvable. They work by penetrating the stratum corneum of the skin to create hundreds of reversible microchannels without causing skin damage or pain (Figures 6A and B). Microneedles provide safe passages for therapeutic substances, especially large molecules, to bypass barriers and deliver various therapeutic compounds.^198^

Wan et al.^15^ reported a microneedle patch that mediates the synergistic transdermal delivery of CRISPR/Cas9‐based genome editing agents and glucocorticoids for the effective treatment of inflammatory skin diseases. The results showed the microneedle patch system could degrade rapidly in vivo after penetrating the skin to release therapeutic substances. Blood biochemical functional indices have also validated that microneedle patch‐mediated therapy has minimal systemic toxicity and is safe for clinical use.^15^ Lara et al.^199^ further demonstrated that siRNA delivered through microneedle arrays could reduce the expression of the targeted endogenous gene CD44 in human skin xenograft models.

The use of solid, coated, lysis, or hollow microneedles for gene therapy with nucleic acids as cargo needs to be improved. Microneedles alone may not facilitate the delivery of genetic material better than intradermal injections, but the combination of microneedles and electroporation is more effective than injections alone. The combination of microneedles with other delivery techniques may therefore also be effective. Compared with other physical delivery vehicles that have disadvantages such as skin damage, high manufacturing costs, and not being preferred by patients, microneedles perform well in these aspects. Moreover, various bioactive ingredients can be added to the formulations of dissolvable microneedles to enhance their effects. Microneedles are also painless when used and, therefore, better for DNA‐based vaccinations than other forms of immunization that require multiple injections and cause pain for days or even weeks.^198^

### 5.2 Microspheres

Microspheres are flowable spherical particles that can be loaded with specific substances. They are also biocompatible and biodegradable. Their particle sizes range from 1 to 250 microns. There is a wide variety of microsphere formulations that can be classified into three categories according to their structure: pore‐forming, double‐layered, and magnetic microspheres. Commonly used methods for microsphere preparation include evaporation of emulsification solvent, phase separation, spray drying, electro‐spraying, and microfluidic methods.^200^, ^201^ Different manufacturing processes can produce microspheres with different structural characteristics to allow microspheres to be used in different medical applications.^202^

Materials for the preparation of microspheres include glass (such as that made of silicate, borate, or phosphate), ceramics, and polymers. Polymer‐based porous microspheres have been extensively investigated for drug release and as delivery vehicles for other biological components. Polymer‐based microspheres are mainly classified into natural polymeric microspheres (e.g., proteins, collagen, chitosan, and alginate) and synthetic polymeric microspheres (e.g., PLGA, PLA, PGA, and PCL) that can be hydrolytically degraded in vivo.^201^ Diez et al.^203^ prepared different PLGA microspheres encapsulating DNA using two formulation schemes. The results showed that the formulations, molecular weights, and compositions of the polymers used to prepare the particles are important determinants of the sizes and encapsulation and release behaviors of these delivery systems (Figure 6C). These microspheres have other advantages, as they can deliver plasmid DNA at a rapid and controlled rate.^203^

Microspheres are excellent vehicles for the establishment of oral nucleic acid delivery systems. They can protect the encapsulated material during transport through the gastrointestinal tract and facilitate efficient uptake and intracellular transport to the desired target site, while being safe and well tolerated. Attarwala et al.^204^ developed a multicompartment system based on an oral system of nanoparticles within microspheres for in vivo gene and siRNA delivery to treat colitis in mice (Figure 6D). The results exhibited effective transgene expression or gene silencing, as well as downstream anti‐inflammatory effects.^204^ In addition, Guo et al. designed microsphere systems that could slowly release loaded substances locally and inject them into intervertebral discs to exert sustained therapeutic effects. This microsphere‐based delivery system can alleviate low back pain, inhibit tissue degeneration, and promote disc regeneration or repair by targeting the delivery of cells, drugs, bioactive components, and genetic modifiers to the interior of the disc tissue.^205^

There are also limitations to the use of microspheres. When designing and developing new microsphere formulations, it is difficult to reproduce or predict the full impact of the human environment on injectable microspheres, including changes in pH, temperatures, and enzymes.^206^ Synthetic polymeric microspheres have been proven to be safe and biocompatible, but migrate from the injection site, which leads to potential risks of embolism and further organ damage.^200^

### 5.3 Hydrogels

**FIGURE 7:**
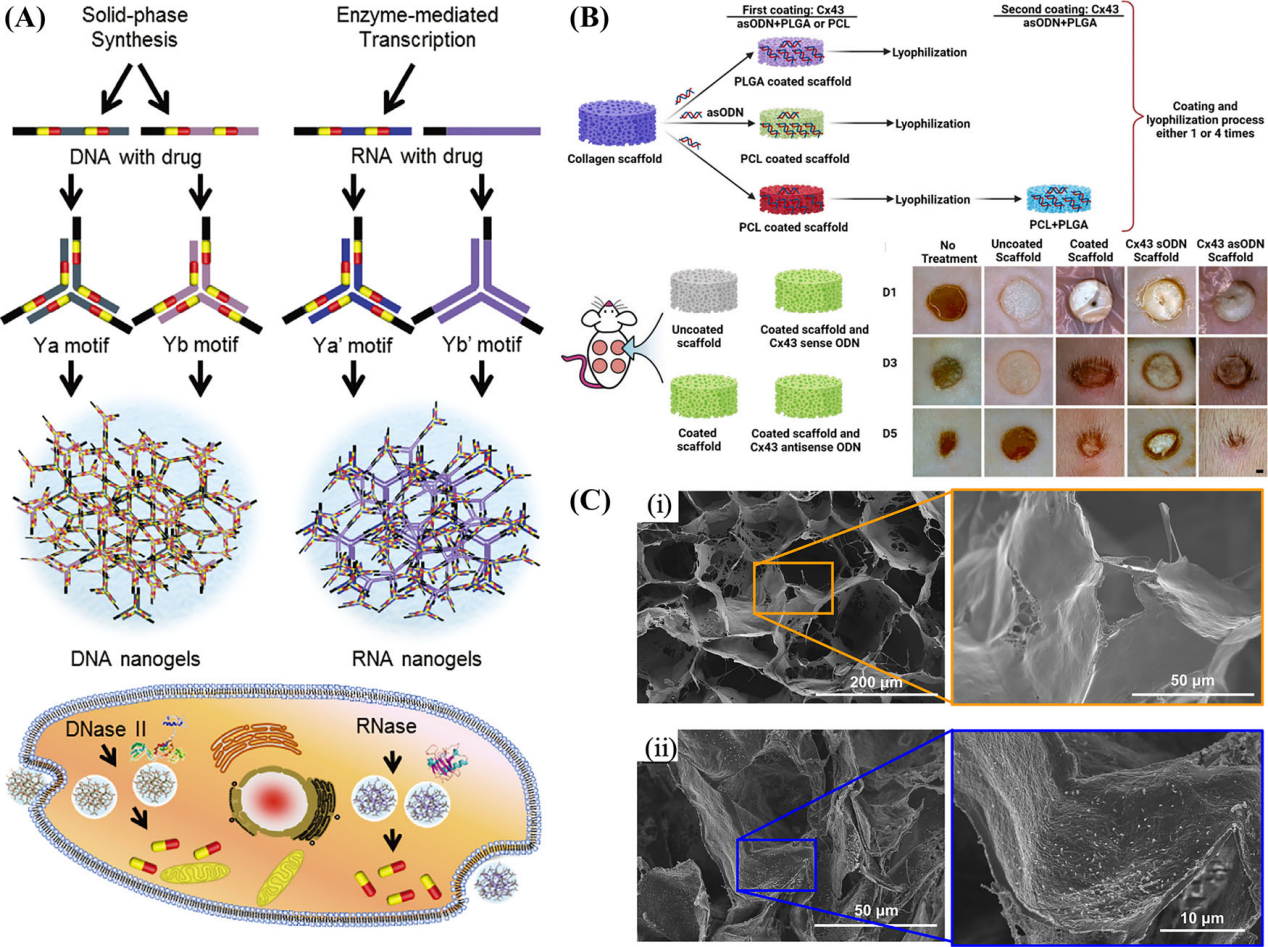
(A) DNA hydrogel‐based delivery system for small‐molecule drugs. Reproduced with permission from Ref. 212, Copyright 2021 © Springer Nature Limited. (B) Schematic diagram of monolayer and multilayer scaffolds. Reproduced with permission from Ref. 288, Copyright 2022 © Elsevier B.V. (C) Visualization of PEI‐pIL‐1Ra nanoparticles in a collagen‐hydroxyapatite scaffold. (i) SEM image of the porous microarchitecture in CHA scaffolds. (ii) SEM image of a CHA scaffold activated with PEI‐pIL‐1Ra nanoparticles. Reproduced with permission from Ref. 225, Copyright 2020 © Lackington, Gomez‐Sierra, González‐Vázquez, O'Brien, Stoddart and Thompson.

Hydrogels may be formed through the polymerization of small molecules with numerous hydrophilic groups. Due to their water‐rich composite structure and the cross‐linking of long polymer chains, hydrogels exhibit elastic, adhesive, and mechanical properties that make them suitable as biomaterials.^207^, ^208^ Hydrogels can be classified into three main categories according to their size: macro/bulk hydrogels, microgels, and nanogels (Figure 7A).^209^ DNA hydrogels at the micro‐ or nanoscale are more responsive to stimuli and have enhanced delivery efficiencies. Based on the origins of the monomers, hydrogels can also be divided into two categories: naturally occurring hydrogels, such as chitosan, HA, gelatin, collagen, fibrin, and alginate, and synthetic hydrogels, which are based on hydrophilic synthetic polymers such as PAA, polyvinyl alcohol (PVA), PAM, and PEG.^210^, ^211^ DNA hydrogels comprise a hydrophilic polymer network of cross‐linked DNA strands. Unlike conventional vectors, DNA hydrogels can improve cell transfection efficiency, protect biomolecular payloads from nuclease or proteases degradation, and reduce nonspecific distribution.^212^ DNA hydrogels are promising carrier materials that are biocompatible, simple to prepare, have tunable mechanical properties, and allow for a controllable phase transition. In addition to enabling the in situ encapsulation of drugs, DNA hydrogels allow for molecular recognition within target regions and the integration of multiple components for synergistic therapy.^213^ In 1996, Nagahara and Matsuda^214^ designed the first DNA‐based hydrogel by cross‐linking single‐stranded DNA grafted onto polyacrylamide chains. Considering that DNA strands are programmable, complementary, and chemically modifiable, they can be manipulated to form various DNA building blocks with unique geometries and form highly predictable and structured DNA networks. In addition, 3D scaffolds in DNA hydrogels provide mechanical rigidity and numerous attachment sites, enhancing the function of hydrogels as stable immobilization matrices for bound nanoparticles or molecular components.^212^ Due to their biocompatibility, porosity, sequence programmability, and tunable versatility, DNA hydrogels have been extensively studied in bioanalyses and biomedicine.^212^

Recently, Song et al.^215^ designed a thermosensitive hydrogel loaded with a gene complex and implanted it into a postoperative cavity to inhibit the immune escape of residual tumor cells after surgery. A novel nonviral vector, G5‐BGG, was synthesized and formed a gene complex with an shRNA plasmid. A PLGA–PEG–PLGA hydrogel loaded with G5‐BGG/shRNA871 and combined with temozolomide caused the downregulation of CD47 protein expression, increased macrophage infiltration into residual tumors, and significantly prolonged the survival of mice. This study demonstrates the potential use of hydrogel vectors in gene therapies for glioblastoma.^215^ Hydrogels possess good flexibility and biocompatibility, although their mechanical properties are weak. Additionally, conventional hydrogels lose their original properties and suffer irreversible damage when they are damaged or fatigued by use, thus severely limiting the expansion of hydrogel applications. Therefore, there is a requirement for hydrogels with excellent deformability. As a functional hydrogel that can repair itself after damage, self‐healing hydrogel can be realized through external stimulation (light, heat, pH adjustments, and self‐healing agents) or the interaction of functional groups within the hydrogel (dynamic covalent bonding, noncovalent bonding interaction).^216^ The inflammatory response to self‐healing hydrogels is relatively weak.^217^ Chen et al.^218^ constructed hydrogels of aldehyde HA (HA‐CHO) and poly(amidoamine) PAMAM/siRNA complexes. This novel injectable self‐healing hydrogel was found to have significantly alleviated disc inflammation while slowing disc degeneration by efficiently and stably silencing IFN gene expression in nucleus pulposus cells.^218^

### 5.4 Scaffolds

Scaffold‐based systems are effective methods for improving the efficiency of CRISPR/Cas9 targeting gene editing delivery systems. Ho et al.^219^ proposed an innovative scaffold‐mediated CRISPR/Cas9 delivery system by anchoring the CRISPR/Cas9 complex on the surface of an injectable scaffold intended for local delivery. LNP‐Cas9‐RNPs and chemokine CXCL12α were loaded onto a mesenchymal stem cell membrane‐coated nanofiber (MSCM‐NF) scaffold that mimicked the bone marrow microenvironment, targeting the interleukin (IL)‐1 receptor accessory protein (IL1RAP). Continuous local delivery of the Cas9/IL1RAP sgRNA via the CXCL12α‐loaded LNP/MSCM‐NF scaffolds provided an effective strategy to attenuate LSC growth to improve the treatment of acute myeloid leukemia.^219^

Scaffold‐based sustained‐release drug delivery systems not only provide long‐lasting effects but also minimize the risk of toxicity during systemic application (Figure 7B). Short durations and inefficient deliveries have been the main limitations of siRNA due to its sensitivity to nucleases and poor intracellular cytoplasmic delivery efficiency.^220^ Nelson et al. uniformly doped pH‐responsive polymeric micellar nanoparticles in porous, noncytotoxic, biodegradable, and injectable polyester polyurethane (PU) scaffolds to optimize siRNA delivery. siRNAs are depleted in 1 week in rapidly dividing cells,^221^ and they have also been found to be released through diffusion‐based mechanisms across 3 weeks. These findings demonstrate the durable effects and controlled release potential of scaffolds.^222^

Scaffold‐based gene therapy offers a promising approach to tissue engineering as it allows for the transfection of cells to enhance the sustained expression of target proteins or silencing of target genes associated with bone and joint diseases.^223^ Costard et al.^224^ used MgAl‐NO_3_ layered double hydroxide loaded with miRNA nanoparticles and doped them into collagen‐nanohydroxyapatite scaffolds. This resulted in the successful overexpression of target miRNA in mesenchymal stromal cells (MSCs) and demonstrated an effective miRNA delivery platform for gene therapies in regenerative medicine.^224^ Collagen‐hydroxyapatite scaffolds can also be used as platforms for the delivery of nanoparticles composed of plasmid DNA. Such scaffolds have been found to facilitate induction of transient gene expression in rat bone marrow MSCs (BM‐MSCs) through PEI–plasmid DNA nanoparticles encoding the IL‐1 receptor antagonist. These scaffolds improved the mineralization of the BM‐MSCs and demonstrated their potential for future therapeutic applications in vivo (Figure 7C).^225^

## 6 APPLICATIONS OF BIOMATERIAL‐BASED GENE DELIVERY SYSTEMS

### 6.1 Genetic vaccines

The recent COVID‐19 pandemic caused RNA‐based therapies and vaccines for infectious diseases to be brought to the forefront of research worldwide.^34^ The pandemic further led to the marketing of the first approved mRNA vaccine. The COVID‐19 BNT162/Comirnaty vaccine from BioNTech/Pfizer and the COVID‐19 mRNA‐1273/Spikevax vaccine from Moderna were both approved by the US FDA for emergency use in 2020 and re‐approved in 2021 and 2022, respectively.^226^ RNA vaccines against hepatitis B, influenza, and respiratory syncytial viruses have also been developed and are undergoing clinical trials.^34^

**FIGURE 8:**
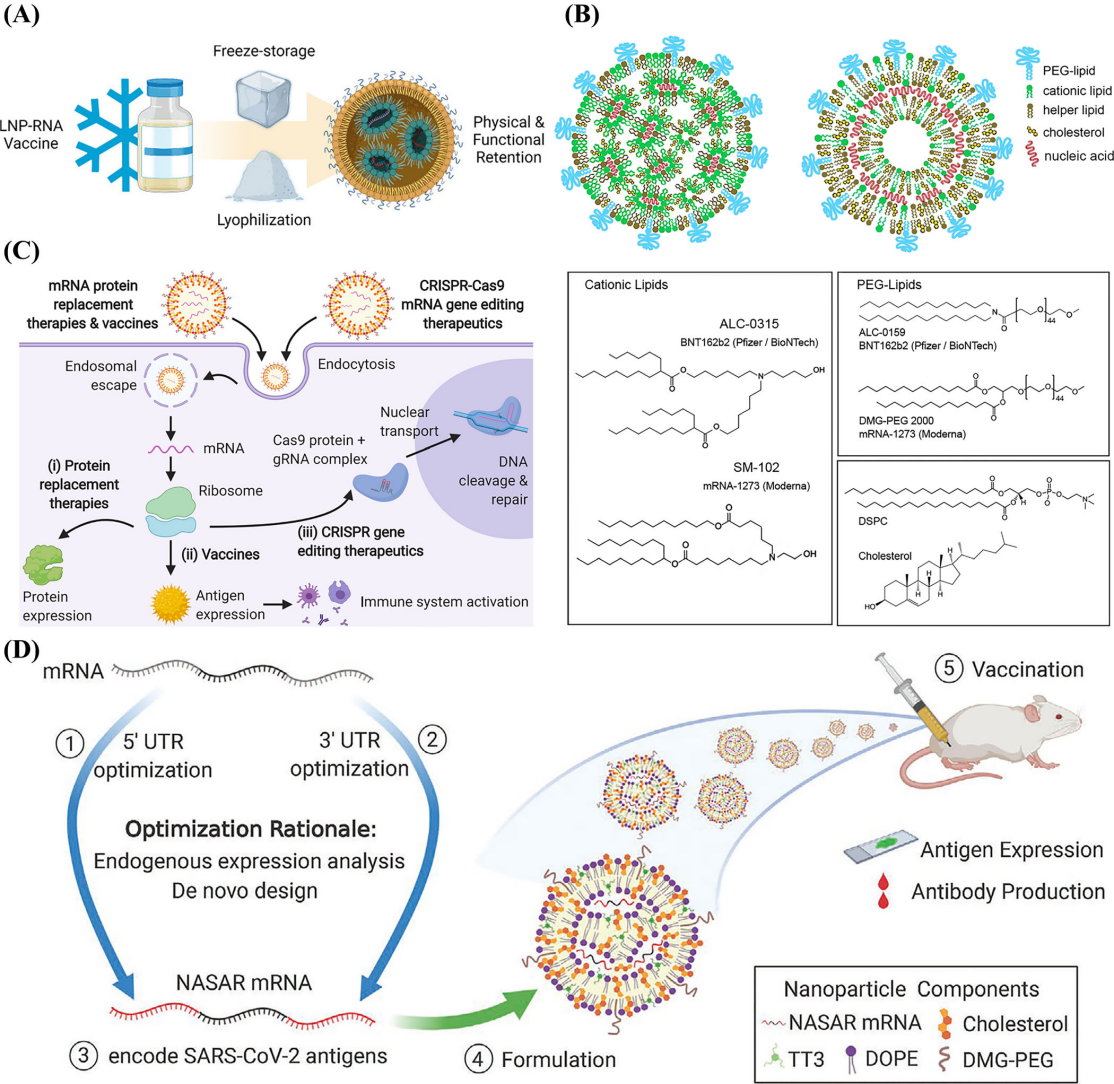
(A) Physical and functional retention of LNP‐RNA vaccine. Reproduced with permission from Ref. 231, Copyright 2022 © The Authors. Published by Elsevier B.V. (B) Structures of lipid nanoparticle nucleic acid vectors. Reproduced with permission from Ref. 289, Copyright 2021 © The Authors. Published by American Chemical Society. (C) The mRNA is encapsulated in lipid nanoparticles. Reproduced with permission from Ref. 227, Copyright 2021 © Elsevier Ltd. (D) mRNA engineering process used as a SARS‐CoV‐2 vaccine. Reproduced with permission from Ref. 228, Copyright 2020 © Wiley‐VCH GmbH.

LNPs are the most widely used nonviral delivery systems for nucleic acid vaccines.^34^ The delivery of RNA to target cells via LNPs allows to treat various diseases, including COVID‐19.^227^ The composition of LNPs in COVID‐19 vaccines includes ionizable lipids, cholesterol, helper lipids, PEG2000 lipids, and mRNA.^228^ However, the excipient, PEG, may cause adverse effects. PEG has been shown to trigger allergic reactions, including anaphylaxis and contact urticaria, in some vaccine recipients.^229^ Therefore, alternative chemicals to PEG should be identified to improve mRNA vaccine vectors or develop new delivery vehicles based on polymers or protein particles, such as protamine. Storage and transport in the form of lyophilized powder is the most commonly used method for mRNA drugs. For mRNA vaccines encapsulated in liposomes, the addition of sucrose can reduce the crystallization temperature and the amount of ice formation during the aqueous phase. Furthermore, the interfacial film formed by the adsorption of sugar molecules at the oil‐water interface can also increase the spatial potential resistance and electrostatic repulsion between droplets, which can in turn improve their low‐temperature and mRNA stability. The addition of cryoprotectants (such as alginate, sucrose, and mannitol) to the mRNA‐LNP solution and storage in liquid nitrogen can ensure the efficiency of an optimal mRNA delivery system in the long term^230^, ^231^ (Figure 8).

Vaccination is an important way to control and eliminate potentially infectious diseases and cancers. Traditional live attenuated vaccines have been used for a long time, but serious safety concerns regarding toxicity and the risk that pathogens mutate back to an infectious state have limited their use.^232^, ^233^ Nucleic acid vaccines have numerous advantages. For example, they avoid the problems associated with recombinant protein vaccines, such as improper protein folding or high protein purification costs. They are also free from the infection risks associated with attenuated or inactivated vaccines that produce self‐activating infectious organisms. Nucleic acid vaccines can also activate humoral and cellular immune responses, greatly enhances the protective immune response. Furthermore, the efficiency and stability of mRNA transactivation can be improved by various chemical modifications, increasing immunity.^234^ Biomaterial‐based antigen delivery systems have emerged as innovative strategies to improve the efficacy of genetic vaccines. Antigen delivery systems are divided into two broad categories: one is based on microparticle‐based delivery systems, including microparticles and nanoparticles, and the other involves hydrogel‐ and scaffold‐based delivery systems, such as solid implants, scaffolds, and hydrogels.^235^, ^236^ Depending on the specific properties of the delivery system being used, different functions can be performed. For example, they may ensure stability by maintaining antigen release and/or by providing reservoirs at the injection site, protecting materials from degradation, and delaying the antigen clearance from the injection site.

### 6.2 Gene therapy for clinical diseases

Gene therapy is widely used in the treatment of clinical diseases, and in this section, we summarize the gene therapy strategies based on biomaterial vectors in recent years (Table 2).

**TABLE 2:** List of biomaterial‐based gene therapy.

Delivery system	Types of bioactive compound	Target gene(s)	Applications	References
PLGA NPs	shRNA	BCH‐BB694 BCL11A	Sickle cell disease	^238^
PEGylated glycosaminoglycan (GAG)‐modified t‐Boc chemosynthetic peptide carriers	DNA	N/A	Cystic fibrosis, asthma, mucosal obstructive lung diseases	^112^
Hyaluronic acid (HA), dextran, and polyvinylpyrrolidone (PVP)‐17 dissolving microneedle	siRNA	STAT3	Melanoma	^244^
PEG–PBAE polyplexes	DNA	HSV‐tk	Small cell lung cancer	^246^
HA‐SeSe‐COOH@PAMAM	siRNA	KRAS	Non‐small cell lung carcinoma	^167^
Protamine sulfate with distearoyl‐phosphoethanolamine‐PEG‐HA	pDNA	MTH1	Non‐small cell lung cancer	^248^
PU short‐branched PEI (PU‐PEI)	miRNA	Oct4, Sox2, and Fascin1	Non‐small cell lung cancer	^249^
Self‐assembled RNA‐triple helix hydrogel	miRNA and siRNA	Trpm1, E2F1, LAMC1 and CXCR4	Breast cancer	^251^
Cyclam‐Modified PEI	siRNA	CSCR4/SDF‐1	Breast cancer	^137^
Oleylamine (OA)‐modified disulfide‐containing PEI	siRNA	Survivin	Liver cancer	^253^
Cationic nanobubbles (CNBs) conjugated with an A10‐3.2 aptamer	siRNA	TK‐p53‐NTR	Hepatocellular carcinoma	^254^
A cavity‐injectable NP hydrogel superstructure	pDNA	CD133‐specific CAR	Glioblastoma	^260^
Demineralized bone matrix (DBM)/MVs‐PEI	pDNA	hBMP2	Bone defect repair and regeneration	^262^
Nano‐MgO micelle composites	mRNA and pDNA	α‐Syn	Parkinson's disease	^266^
PEI‐PEG	pDNA	SMN‐1	Spinal muscular atrophy	^267^
Protamine, dextran, or HA‐modified solid LNPs‐PVA	pDNA	HCE‐2	Corneal Inflammation	^270^
iPSC‐MSC exosomes‐loaded thermosensitive chitosan‐based hydrogels	miRNA	TRAM2	Corneal epithelium and stroma regeneration	^271^
Positively charged poly‐Lys (CK30) PEG NPs	DNA	N/A	Inherited retinal diseases	^268^
PAM‐ABP	pDNA	pβ‐SP‐ODD‐VEGF	Acute ischemic heart disease	^273^
Magnetic NPs	miRNA	Notch1, Sirt1, Pten, and Pnuts	Myocardial infarction	^274^
HPAEs	pDNA	COL7A1	Recessive dystrophic epidermolysis bullosa	^275^
PAMAM dendrimers	siRNA	TNF‐α	Psoriasis	^279^
Liposome	DNA	IL17RA	Psoriasis	^280^
Thiolated glycol chitosan (tGC) NPs	siRNA	TNF‐α	Rheumatoid arthritis	^282^
Folate–PEG–CH–DEAE15	siRNA	TNF‐α	Rheumatoid arthritis	^281^
Noncondensed alginate‐based NPs	pDNA	IL‐10	Rheumatoid arthritis	^284^

#### 6.2.1 Genetic and rare diseases

Gene therapies can be used for genetic disorders such as hemoglobin disorders, cystic fibrosis, hemophilia, familial hypercholesterolemia, and lipoprotein lipase deficiencies. Autologous gene therapies for hemoglobin disorders are especially rapidly advancing. Β‐hemoglobinopathies, such as sickle cell disease and β‐thalassemia, are the most common monogenic disorders caused by mutations in the β‐globin gene, which encodes the two subunits of adult hemoglobin (HbA and α2β2).

Sickle cell disease is an inheritable disorder of the molecular function of hemoglobin. Sickle hemoglobin (HbS) is a variant of normal adult hemoglobin. Upon deoxygenation, HbS polymerizes, resulting in abnormally shaped red blood cells and variable downstream clinical sequelae, including painful vaso‐occlusive crises, chronic hemolytic anemia, progressive and irreversible organ damage, decreased quality of life, and early death.^237^ The ex vivo gene editing of CD34+ hematopoietic stem and progenitor cells (HSPCs) is a good approach for gene therapy. Cruz et al. developed CRISPR/Cas9‐PLGA nanoparticles that efficiently encapsulate the Cas9 protein, sgRNA, and a fluorescent probe. The CRISPR/Cas9‐PLGA‐nanoparticle‐mediated gene editing of the γ‐globin locus was found to cause the elevated expression of fetal hemoglobin (HbF) in primary erythrocytes. HbF was also shown to reduce fetal hemoglobin expression by attenuating HbS aggregation and erythrocyte sickle cell disease to significantly improve sickle cell disease.^238^ Encapsulation of nanoparticles saves the Cas9 and sgRNA from degradation.^239^, ^240^ The nanoparticle core further imparts physicochemical properties to the cargo, enabling more efficient uptake of CRISPR components by the cellular endocytic mechanisms. The development of CRISPR/Cas9‐PLGA nanoparticles has provided an attractive tool to deliver CRISPR components to targeted HSPCs and may provide a basis for in vivo treatments of hemoglobinopathies and other genetic diseases.^241^

Cystic fibrosis is an autosomal recessive disorder that may be caused by mutations in the cystic fibrosis transmembrane conductance regulator gene. Overcoming the airway mucus barrier has been identified as one of the greatest challenges for successful respiratory gene therapy.^242^ Respiratory diseases are characterized by a continuous cycle of lung infection and inflammation, leading to airway mucus obstruction. A wide variety of carrier molecules, such as cationic lipids or polymers, have been developed for applications of gene therapy in cystic fibrosis.^243^ Osman et al.^112^ prepared PEG‐coated and glycosaminoglycan‐modified solid phase tert‐butoxycarbonyl (t‐Boc) chemosynthetic peptide carriers directly conjugated to DNA through electrostatic interactions to form nanoparticles to treat cystic fibrosis, asthma, and mucosal obstructive lung diseases such as chronic obstructive pulmonary disease.

#### 6.2.2 Neoplasms

Melanoma is a serious malignant tumor that originates from melanocytes. Melanoma has poor prognoses, aggressive local growth, high metastatic and mortality rates, is resistant to conventional radiotherapy, and difficult to cure. Transcriptional signal transfer and activator 3 (STAT3) is associated with malignant behaviors such as tumor proliferation, metastasis, angiogenesis, survival, and immune evasion and is highly active in malignancies, including breast cancer, prostate cancer, and melanoma. Thus, siRNA‐based gene therapies targeting STAT3 are potential strategies to treat melanoma. A team of researchers has developed a safe and novel lysis microneedle for the topical application of STAT3 siRNA to enhance skin penetration of the siRNA. The microneedle uses PEI as a carrier to promote siRNA uptake by target cells.^244^

Lung cancer is a fatal malignancy that originates in the bronchial mucosa or glands. It can be classified as small cell lung cancer (SCLC) and non‐small cell lung cancer (NSCLC). SCLC is an aggressive neuroendocrine tumor with a high recurrence rate, limited treatment options, and poor prognoses.^245^ A study by Kim developed block copolymers comprising PBAE oligomeric central units and PEG terminal units to deliver DNA plasmids. The PEG–PBAE complex combined with ganciclovir was found to significantly increase the efficiency of destroying human SCLC cells (H446).^246^ NSCLC is the most common type of lung cancer in humans^247^ and includes squamous cell carcinoma, adenocarcinoma, and large cell carcinoma. Conventional treatments include chemotherapy, radiation therapy, and surgery. Chen et al.^167^ constructed a novel HA‐modified redox‐sensitive hybrid nanocomplex. The efficacy of NSCLC therapy was enhanced by efficient delivery of siRNA to tumor cells through redox‐mediated intracellular disassembly.^167^ Furthermore, Wang et al.^248^ prepared the multifunctional nonviral vector, PS@HA‐Lip, which could actively target tumor cells and deliver CRISPR/Cas9 plasmids to the nuclei of the cells. In addition, it inhibited the growth and induced apoptosis of NSCLC cells by disrupting MTH1 gene expression and reducing liver metastasis associated with NSCLC. This multifunctional vector contained a NLS of protamine sulfate and was modified with distearoyl‐phosphoethanolamine‐PEG‐HA to endow the vector with the ability to actively target tumor cells.^248^ In addition, the highly aggressive and frequent recurrence of lung adenocarcinoma (LAC) is one of the main causes of failed treatments and poor prognoses. Chiou et al.^249^ used PU short‐branched PEI (PU‐PEI) as a vector to deliver miR145 to LAC cancer stem cells (LAC‐CSCs). The PU‐PEI‐mediated delivery of miR145 reduced tumor growth and metastasis and improved the radiotherapeutic resistance of the LAC‐CSCs by directly targeting the transcription factors Oct4, Sox2, and Fascin1, sensitizing the tumors to radiotherapy.

The incidence of breast cancer is the highest among female malignancies worldwide.^250^ Ding et al.^251^ developed a novel RNA‐triple‐helix hydrogel for the treatment of triple negative breast cancers (TNBCs). The RNA‐triple‐helix consists of one tumor suppressor miRNA (miRNA‐205) and one oncomiR inhibitor (miRNA‐221). The siRNA duplexes of CXCR4 were embedded into the RNA hydrogel to block breast cancer metastasis. Self‐assembly of RNA‐triple helix hydrogels exhibits high selectivity for uptake and control of miRNA expression in vitro and in vivo. This gene delivery system provides a potential therapeutic approach with high specificity and selectivity for TNBCs.^251^ The death of most breast cancer patients can be attributed to the metastatic spread of invasive breast cancer.^252^ Zhou et al.^137^ demonstrated that Cyclam‐Modified PEI (PEI‐C) as a carrier can simultaneously silence Vascular endothelial growth factor (VEGF) and inhibit CXCR4 expression, therefore possessing good antitumor and antimetastatic activities.

Gene therapy strategies for liver cancer are promising, but there is still a lack of a stable and efficient vector. To overcome this obstacle, researchers have developed novel biomaterials as carriers for gene delivery. Zhao et al.^253^ designed a novel multifunctional gene delivery vector, sTPssOLP, which is composed of PEI derivatives and cationic lipids bound to siRNA. The modified PEG and transferrin (Tf) are partially encapsulated in a phospholipid bilayer via lipids, and the other part acts as an outer layer. sTPssOLP targeted delivery of siRNA to HCC cells, leading to downregulation of mRNA levels and inhibition of survivin protein expression without damaging normal tissues.^253^ The multistep preparation of PEI, PLGA, and PEG as triblock copolymers by Sukumar et al.^254^ effectively reduced the toxicity of PEI without compromising its transfection efficiency. In vivo delivery of a rationally engineered thymidine kinase (TK)‐p53‐nitroreductase (NTR) triple therapeutic gene using SP94 peptide‐functionalized PLGA–PEG–PEI nanoparticles can effectively treat against HCC.

Most brain diseases are incurable and have high morbidity and mortality rates. Glioblastoma is the most common central nervous system (CNS) malignancy with a short median survival time of 4–15 months after diagnosis^255^ and a 5‐year survival rate of less than 5%.^256^ In addition, the incidence of metastatic brain tumors is over 10 times that of glioblastomas.^257^ Glioblastoma multiforme (GBM) is the most malignant CNS tumor. Surgical resection is the most basic intervention in the clinical treatment of GBM, and a combination of radiotherapy and chemotherapy is used to remove residual tumor cells after surgery. However, glioma stem cells (GSCs), which are known as the “seed cells” of brain tumors, are insensitive to radiotherapy and chemotherapy and are difficult to remove.^258^ After surgical resection of solid tumors in patients with GBM, residual GSCs quickly recover, proliferate, and differentiate, leading to the recurrence of the tumors within a few months.^259^ Therefore, exploring effective strategies for the specific removal of GSCs is crucial to prevent the recurrence of GBM after surgery. A cavity‐injectable nanoparticle hydrogel superstructure that generates GSC‐specific CAR macrophages/microglia (MΦs) around the lumen has been reported to treat GBM. These CAR‐MΦs were found to seek, phagocytose, and remove residual GSCs to prevent GBM recurrence.^260^

#### 6.2.3 Bone defect repair and regeneration

Bone formation and regenerative therapies require optimization and improvement, as many skeletal disorders remain insufficiently treated. For example, clinical solutions for osteogenesis‐related nonunion fractures and osteoporotic vertebral compression fractures are not yet optimal. Therefore, better treatments should be created.^261^ Gene therapy is one such treatment that has long been studied for the regeneration of missing bone tissue and healing fractures.

The transient gene expression of the bone formation protein (BMP) family members BMP‐2, −4, −6, and −9 has been repeatedly sufficient for bone formation. Although autologous bone grafts are the gold standard treatment for these diseases, they can lead to complications at the donor site. There is also limited availability of autologous bone grafts. Recombinant human BMP‐2 (rhBMP‐2) and rhBMP‐7 are the only biological solutions available to avoid the need for bone grafts.^261^ BMP treatments require large doses of protein—up to 1.5 mg of protein/mL of matrix—and are therefore not cost effective. In addition, gene therapies targeting recombinant human bone‐forming proteins have been extensively studied to avoid the side effects and complications of direct rhBMP treatments and improve bone regeneration methods. BMP‐6 and ‐9, followed by BMP‐2, have also been found to be the most effective inducers of osteogenic differentiation among 14 BMP genes.

Nonviral vectors are attractive alternatives to viral vectors as they elicit minimal immune responses in the host. When the aim of a therapy is bone formation, transient gene expression, which is the expression achieved with nonviral vectors, is desirable as it limits the amount of newly formed bone and reduces the chances of malformation. In recent years, plasmid DNA containing therapeutic genes coupled with biodegradable scaffolds have been fabricated as gene‐activated matrices (GAMs). GAMs are implanted in areas of interest, such as ectopic or defective sites, allowing the slow delivery of therapeutic DNA to surrounding cells to increase the efficiency of bone formation.^261^ Liang et al.^262^ obtained BM‐MSC‐derived MVs. PEI and human bone morphogenetic protein 2 (hBMP2) plasmid were then sequentially encapsulated in then MVs through layer‐by‐layer self‐assembly to form the nonviral gene vector MVs‐PEI/phBMP2. They were then loaded onto demineralized bone matrix (DBM) scaffolds to fabricate gene‐activated scaffolds called “DBM/MVs‐PEI/phBMP2.” MVs‐PEI/phBMP2 exhibited a higher transfection efficiency and lower cytotoxicity to MSCs, enhanced the osteogenic differentiation of MSCs in vitro, and promoted angiogenesis. These findings indicated that this gene‐activated scaffold may be a promising bone replacement material.^262^ Biomaterial‐based nanoparticles have been extensively studied for the delivery of therapeutic factors that promote bone regeneration or formation in vivo or in vitro.^94^ Calcium phosphate is one of the most promising materials for bone repair due to its osteoconductivity, osteoinductivity, and ability to be resorbed in vivo. Plasmid DNA, miRNA, and siRNA can be easily bound to the surfaces of calcium phosphate nanoparticles. Calcium phosphate nanoparticles packed with therapeutic factors have also been delivered to bone injury sites using scaffolds and hydrogels.^263^

#### 6.2.4 Neurodegenerative diseases

Gene therapies targeting the brain have emerged as promising strategies to treat neurological disorders.^264^ Considering the blood–brain barrier (BBB), brain‐targeted gene therapy requires high‐quality nonviral vectors. Patients with Parkinson's disease (PD) routinely receive levodopa to restore dopaminergic transmission and improve motor‐related symptoms, such as bradykinesia, rigidity, and tremors. However, levodopa becomes less effective over time and may cause dyskinesia and disability. Therefore, the development of gene therapy vectors for PD is an important direction for future research. PD occurs due to oxidative stress, mitochondrial aberrations, posttranslational modifications, and α‐synuclein (α‐Syn) aggregation.^265^ Despite the remarkable results of gene therapies targeting the α‐Syn gene in the treatment of PD, the lack of suitable gene delivery systems and insufficient therapeutic efficacy remain huge obstacles for RNAi‐based therapies. Li et al.^266^ constructed a degradable nano‐MgO micelle composite (MgO(plasmid DNA)‐INS‐Plu‐mRNA‐NGF) with double interference mediated by RNAi and α‐Syn‐targeted mRNA. The complex penetrated the BBB and transmitted genes and mRNA to neurons through nerve growth factor and endocytosis mediated by its receptor, which significantly downregulated the expression of α‐Syn in PD mouse models without causing damage to other major organs.^266^

Spinal muscular atrophy (SMA) is caused by a gene mutation in the survival motor neuron (SMN)‐1 gene, which results in a dramatic decrease in the level of SMN proteins. The delivery of genes to the spinal cord provides a potential approach to treat spinal cord trauma, amyotrophic lateral sclerosis, and SMA. These diseases progress over time and require stable expression of functional genes at therapeutic levels for months or years. Shi et al.^267^ explored the feasibility of achieving extended transgene expression in the spinal cords of rats by repeated intrathecal administration of plasmid DNA complexed with 25‐kDa PEI into the subarachnoid cavity of the lumbar spine. The DNA/PEI complex was found to provide 40‐fold higher transgene expression in the spinal cord than the naked plasmid DNA could through a single injection. PEI was also found to be modified with PEG, which reduced the attenuation of gene expression.^267^

#### 6.2.5 Ophthalmic diseases

Gene therapies for eyes have been widely explored as a therapeutic route to target diseases of the cornea, retina, and RPE.^268^ Nonviral gene therapy can provide safer and more effective options for treating IRDs.^269^ Vicente‐Pascual et al.^270^ used protamine, dextran, and HA and formulated a nonviral vector based on solid LNPs and PVA as a gene delivery system for the induction of IL‐10 expression. Eye drops developed in this study may be of use in the treatment of corneal inflammation.^270^ For the treatment of corneal injury, Tang et al.^271^ developed a new approach based on induced pluripotent stem cell‐derived mesenchymal stem cells (iPSC‐MSC) combined with thermosensitive hydrogel exosomes, which reduce scar formation and accelerate the healing process. iPSC‐MSC exosomes can effectively promote the repair of damaged corneal epithelium and stroma by downregulating the expression of mRNAs encoding the three most enriched collagen proteins (collagen type I α1, collagen type V α1, and collagen type V α2). In addition, miR‐432‐5p could prevent ECM deposition through a mechanism related to the suppression of the target gene TRAM2.^271^ The RPE performs various critical functions necessary to maintain the neural retina and proper vision. Furthermore, the tight junctions between RPE cells form the blood‐retinal barrier and help the eye maintain its immune privileges. Many blinding retinal diseases, including Leber's congenital amaurosis and retinitis pigmentosa, occur due to mutations in RPE‐specific genes. Choroidal neovascularization, age‐related macular degeneration, and other retinal diseases are also associated with defects in RPE‐related structures. Although many strategies have been explored, there are no curative therapies for RPE‐associated diseases. The most developed option to treat RPE‐based diseases is gene therapy. One study, for example, conjugated vectors of positively charged poly‐Lys (CK30) with PEG nanoparticles and found that they could improve the expression of therapeutic genes in the retina.^268^

#### 6.2.6 Cardiovascular diseases

Cardiac death due to myocardial infarction results from a lack of an oxygen supply and blockage of tissue coronary arteries. The goal of cardiac gene therapy is essentially to minimize damage and promote regeneration. The safe and efficient transfer of genes into the myocardium is critical for cardiac gene therapy, and nonviral gene vectors comprise one form of treatment studied. Nonviral vectors in cardiac research include lipid‐based, polymer‐based, and cell‐mediated reagents (i.e., cells that have been transfected before implantation).^272^ VEGF‐based gene therapies that promote therapeutic angiogenesis have been developed as an alternative treatment for myocardial ischemia. However, the unregulated expression of VEGF and the use of viral vectors have slowed the clinical development of angiogenic gene therapies. VEGF‐based gene therapies now require disease‐specific gene expression systems and efficient nonviral gene vectors. Won et al.^273^ developed a novel posttranslationally regulated hypoxia‐associated VEGF plasmid (pβ‐SP‐ODD‐VEGF) and a dendritic molecular‐type bioreducible polymer (PAM‐ABP). This system protected cardiomyocytes from apoptosis, preserved left ventricular function, and prevented left ventricular remodeling. Therefore, this therapy was effective to treat acute ischemic heart disease. PAM‐ABP significantly increased the efficiency of transfected VEGF plasmids and resulted in only small areas of collagen deposition. The infarct areas in the PAM‐ABP/pβ‐SP‐ODD‐VEGF‐treated hearts were also significantly smaller.^273^ The CRISPR/Cas9 coupled magnetic nanoparticles developed by Park et al.^274^ were guided into the heart by an external magnetic field. The CRISPR/Cas9 magnetoplexes system targeting miR34a in vivo can improve cardiac repair and regeneration to promote improved cardiac function in myocardial infarction.^274^

#### 6.2.7 Dermatological diseases

Nonviral gene therapies for hereditary skin diseases have attractive prospects; however, research efforts in this field have been rare.^275^ Epidermolysis bullosa is caused by mutations in various genes that encode structurally or functionally related proteins.^276^ Genetically, recessive dystrophic epidermolysis bullosa (RDEB) is caused by loss‐of‐function or biallelic mutations in the COL7A1 gene, which result in the under expression or deletion of collagen type VII. There are no cures available for RDEB. The clinical management of RDEB is limited to symptomatic treatment and skin care aimed at promoting healing, controlling infection, relieving pain and pruritus, and preventing disease complications.^277^ Zeng et al.^275^ developed a gene delivery platform based on highly branched PBAE (HPAE) to treat RDEB. HPAEs have a 3D spatial structure and are believed to improve polymer–DNA interactions, prevent the enzymatic degradation of DNA, and increase the cellular uptake of the complex, acting as safe and efficient nonviral vectors.

Psoriasis is a complex, immune‐mediated, chronic inflammatory skin disease that affects a wide range of epithelial and immune cells.^278^ Local delivery of siRNA in gene therapy for psoriasis is a challenging task due to the complex barrier properties of the stratum corneum. Pandi et al.^279^ applied PAMAM dendrimers loaded with siRNA that encodes tumor necrosis factor (TNF)‐α. IL‐6, TNF‐α, IL‐17, and IL‐22 levels were reduced in a psoriasis plaque model. This indicates that dendrimers can improve skin permeability and serve as potential carriers for local gene delivery.^279^ Furthermore, Liu et al.^280^ used liposomal spherical nucleic acids (L‐SNAs) targeting IL‐17ra in a topical treatment for psoriasis in mice. The results validated the use of locally delivered L‐SNAs as nanoparticle platforms to knock down IL‐17A receptor expression.

#### 6.2.8 Rheumatoid arthritis

Rheumatoid arthritis (RA) is an autoimmune, chronic, and systemic inflammatory disease. Affected patients are prone to fragility fractures secondary to osteoporosis and bone loss. They may also have functional impairments.^281^ Traditional RA medications act slowly and have prominent side effects. Gene therapies for RA have now emerged, including biomaterial‐based nonviral vectors. Among the proinflammatory cytokines associated with the pathogenesis of RA, TNF‐α plays a critical role in the release of other cytokines and the induction of chronic inflammation. Despite their therapeutic potential, siRNAs are difficult to deliver to target cells because of their poor stability in physiological fluids. Lee et al.^282^ designed polymeric siRNA/thiolated glycol chitosan (psi‐tGC) nanoparticles targeting TNF‐α to treat RA. Compared with methotrexate (5 mg/kg), intravenous injections of psi‐tGC nanoparticles remarkably inhibited inflammation and bone erosion in mice with collagen‐induced arthritis. Therefore, psi‐tGC‐based therapy targeting specific cytokines may produce a new era of RA treatments.^282^ In addition, Shi et al.^281^ developed folate–PEG–CH–DEAE15, which is a chitosan (CH)‐modified nanocarrier deploying folic acid, diethyl ethylamine (DEAE), and PEG, to deliver siRNA to silence the expression of TNF‐α and treat RA. The gene carrier protected siRNA from being damaged by nucleases, and its ligand promoted siRNA uptake through cell surface receptors. The vector further improved solubility at a neutral pH and translocated its load into target cells. The folate–PEG–CH–DEAE15/siRNA nanoparticles did not alter cell viability and significantly reduced inflammation, articular cartilage destruction, and bone loss.^281^ The severity of RA is correlated with the number of macrophages in the arthritic synovium.^283^ Given that macrophages are activated and amplified in RA, Jain et al.^285^ studied the effectiveness of nonviral gene transfection strategies to repolarize macrophages from M1 to M2 phenotypes to treat RA. Anti‐inflammatory cytokines, such as IL‐10, encoding plasmid DNA, were successfully^284^, ^286^, ^287^, ^288^, ^289^ encapsulated by noncondensed alginate‐based nanoparticles, the surfaces of which were modified with prophagocytic peptides to achieve active macrophage targeting. Therapy significantly reduced the expression of the proinflammatory cytokines TNF‐α, IL‐1β, and IL‐6 in systemic and joint tissues and arrested the progression of inflammation and joint damage. Treatment allowed experimental animals to maintain their mobility throughout the study, whereas the untreated animals exhibited impaired mobility.^285^

## 7 SUMMARY AND OUTLOOK

Gene therapy offers great potential to treat genetic disorders and many refractory diseases, as therapeutic gene agents can manipulate the proteins and molecules involved in signaling pathways. Compared with traditional surgery, radiotherapy, and chemotherapy, precision treatments with gene therapies are more advantageous. Gene therapy can overcome the limitations of traditional small‐molecule drugs which only modulate at the protein level. Gene therapy instead works at the genetic level and provides a unique advantage for targets where the causative gene is clear but difficult to treat with drugs at the protein level. One advantage of gene therapy is that it addresses the underlying problem and has the potential to treat the disease in a one‐time manner. Compared with broad‐spectrum small‐molecule drugs, gene therapy has at well‐defined targets, with this specificity providing a reduction in systemic toxicity and other possible side effects. With the rapid development of gene sequencing and transcriptomics, alongside our increasing understanding of the genetic mechanisms of disease occurrence, gene therapy is gradually developing to achieve individual precision treatment. In the past few decades, studies on exogenous gene transfer methods have focused mainly on vector‐free physical methods and viral vectors. However, the low efficiency of physical methods and the resultant cell damage and tissue trauma limit their applications in gene delivery. The immunogenicity, cytotoxicity, and potential infection risks of viral vectors also limit their use as gene delivery vectors. Nonviral vectors now represent the future of gene therapy. However, traditional nonviral vectors have been plagued by low transfection efficiencies, short‐term expression, and low expression levels. Although nonviral vectors are advantageous in terms of their safety, relative ease of production, and versatility, their use has not yet been optimized. Therefore, effective vectors capable of transfection of multiple cell types in vitro and in vivo must be developed.

With the advent of surface‐modified and functionalized materials, biomaterial‐based nonviral gene delivery vectors for gene therapy have rapidly evolved. Biomaterials can be categorized as natural or synthetic polymers and cellular derivatives depending on their source. Biomaterial‐based delivery systems are being developed, including liposomes and their derivatives, peptides, polymers of synthetic or natural origin, dendrimers, inorganic nanoparticles, and EVs. Biomaterials can be further reprocessed into microneedles, microspheres, hydrogels, and scaffolds to provide long‐lasting effects and minimize systemic toxicity.

Various aspects of biomaterial‐based gene delivery systems remain problematic, such as the contradiction between loading efficiency and cytotoxicity, inefficient targeted delivery, and industrial manufacturing of biomaterials. Although numerous researchers are developing nonviral vectors based on biomaterials, viral vectors remain the preferred choice for gene therapy. Further research required for biomaterial‐based gene therapy should be used to potentially advance the technology for clinical applications. Research on these systems and their limitations is still in its early stages. However, exogenous gene delivery systems may be developed into “stealthy” nontoxic systems to avoid macrophage and immune system responses, have good pharmacokinetic properties, be able to infiltrate and target specific tissues, and be responsive. Nonviral gene therapy vectors can be further optimized by designing their modularity. Modifications using functional groups and grafting specific ligands to mediate the appropriate interactions between biomaterials and the extracellular environment or specific cells may yield various benefits. Peptide ligands that may be used on the surfaces of nanoparticles include antibodies, antibody fragments, growth factors, peptides such as CCPs and iRGD, and Tf, whereas nonpeptide ligands include aptamers, folic acid, carbohydrates, and polysaccharides. Double ligand modifications comprise a recently developed approach to enhance specific targeting. The development of intelligent delivery systems that can rapidly secrete nucleic acid drugs in target tissues at a specific time and concentration based on environmental cues is currently in progress. The continuous development of structure‐function relationships combined with mathematical models and fundamental research into the functions and cellular processes of biomaterials may further allow the molecular‐scale design of synthetic vectors. Ultimately, effective biomaterial‐based vectors can expand the clinical use of gene therapy. The development of exogenous gene delivery systems based on biomaterials may be an important focal point in future research, and the application of such systems in cell therapy may further reshape various practices in medicine.

## AUTHOR CONTRIBUTIONS

L. J. L. and X. Y. X. offered direction and guidance for the manuscript. Y. Y. and Y. J. G. drafted the initial manuscript and prepared the figures. L. M. H., B. R. F., and W. H. G. revised the figures for the entire manuscript. P. Y. and Y. K. J. revised the framework for the manuscript. All authors have checked the manuscript and agree to be publication.

## CONFLICT OF INTEREST STATEMENT

The authors declare no conflicts of interest.

## ETHICS STATEMENT

No ethical approval was required for this study.

## Data Availability

The data in this study are available upon reasonable request.
